# Neuraminidase 1 secondary deficiency contributes to CNS pathology in neurological mucopolysaccharidoses via brain protein hypersialylation

**DOI:** 10.1172/JCI177430

**Published:** 2025-06-17

**Authors:** TianMeng Xu, Rachel Heon-Roberts, Travis Moore, Patricia Dubot, Xuefang Pan, Tianlin Guo, Christopher W. Cairo, Rebecca J. Holley, Brian Bigger, Thomas M. Durcan, Thierry Levade, Jerôme Ausseil, Bénédicte Amilhon, Alexei Gorelik, Bhushan Nagar, Shaukat Khan, Shunji Tomatsu, Luisa Sturiale, Angelo Palmigiano, Iris Röckle, Hauke Thiesler, Herbert Hildebrandt, Domenico Garozzo, Alexey V. Pshezhetsky

**Affiliations:** 1 Department of Anatomy and Cell Biology, McGill University, Montreal, Quebec, Canada.; 2 CHU Sainte-Justine Research Centre, Department of Paediatrics, University of Montreal, Montreal, Quebec, Canada.; 3 Department of Neuroscience, Faculty of Science, McGill University, Quebec, Canada.; 4 Department of Chemistry, University of Alberta, Edmonton, Alberta, Canada.; 5 Faculty of Biology Medicine and Health, University of Manchester, Manchester, United Kingdom.; 6 Centre for Regenerative Medicine, Institute for Regeneration and Repair, The University of Edinburgh, Edinburgh, United Kingdom.; 7 The Neuro’s Early Drug Discovery Unit, McGill University, Montreal, Quebec, Canada.; 8 Université Toulouse, INSERM, CHU, CRCT, Toulouse, France.; 9 Département de Neuroscience, Université de Montréal, Montréal, Quebec, Canada.; 10 Department of Biochemistry, McGill University, Montreal, Quebec, Canada.; 11 Nemours/Alfred I. duPont Hospital for Children, Wilmington, Delaware, USA.; 12 CNR – Institute for Polymers, Composites and Biomaterials, Catania, Italy.; 13 Institute of Clinical Biochemistry, Hannover Medical School, Hannover, Germany.

**Keywords:** Genetics, Neuroscience, Genetic diseases, Glycobiology, Lysosomes

## Abstract

Mucopolysaccharidoses (MPS) are lysosomal storage diseases caused by defects in catabolism of glycosaminoglycans. MPS I, II, III, and VII, which are associated with lysosomal accumulation of heparan sulphate (HS), manifest with neurological deterioration and currently lack effective treatments. We report that neuraminidase 1 (NEU1) activity is drastically reduced in brain tissues of patients with neurological MPS and mouse models but not in neurological lysosomal disorders without HS storage. Accumulated HS disrupts the lysosomal multienzyme complex of NEU1 with cathepsin A, β-galactosidase (GLB1), and glucosamine-6-sulfate sulfatase (GALNS), leading to NEU1 deficiency and partial GLB1 and GALNS deficiencies in cortical tissues and induced pluripotent stem cell–derived (iPSC-derived) cortical neurons of patients with neurological MPS. Increased sialylation of N-linked glycans in brains of patients with MPS and mice implicated insufficient processing of sialylated glycans, except for polysialic acid. Correction of NEU1 activity in MPS IIIC mice by lentiviral (LV) gene transfer ameliorated previously identified hallmarks of the disease, including memory impairment, behavioral traits, and reduced levels of excitatory synapse markers VGLUT1 and PSD95. Overexpression of NEU1 also restored levels of VGLUT1/PSD95–positive puncta in cortical iPSC-derived MPS IIIA neurons. Our results demonstrate that HS-induced secondary NEU1 deficiency and aberrant sialylation of brain glycoproteins constitute what we believe is a novel pathological pathway in the neurological MPS spectrum crucially contributing to CNS pathology.

## Abstract 2

Xu, Heon-Roberts et al. show that accumulation of a glucosamine heparan sulfate in neurological mucopolysaccharidoses, a spectrum of rare genetic metabolic conditions, causes disruption of the multienzyme lysosomal comlex leading to secondary enzymatic deficiencies of lysosomal glycosidases and pathological oversialylation of brain glycoproteins.

## Graphical abstract

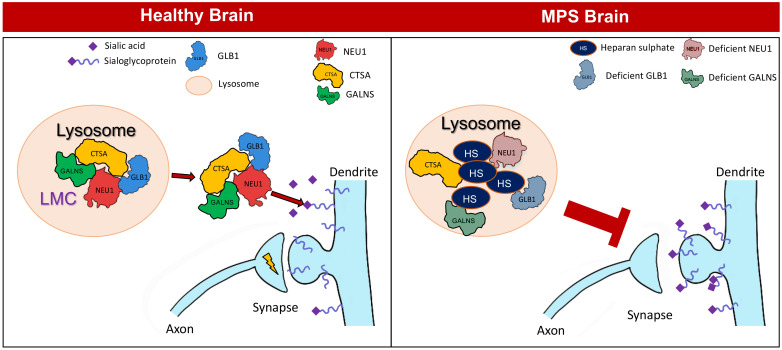

## Introduction

The mucopolysaccharidoses (MPS) are a family of 11 lysosomal storage disorders (LSDs) caused by mutations affecting enzymes involved in degradation of glycosaminoglycans (GAGs) (1). Neurological MPS are characterized by the accumulation of heparan sulphate (HS) caused by a deficiency of α-l-iduronidase (MPS I), iduronate-2-sulfatase (MPS II), iduronate-2-sulfatase (MPS II), *N*-sulfoglucosamine sulfohydrolase (MPS IIIA), *N*-acetyl-α-d-glucosaminidase (MPS IIIB), acetyl-CoA:α-glucosaminide *N*-acetyltransferase (HGSNAT) (MPS IIIC), *N*-acetylglucosamine-6-sulfate sulfatase (MPS IIID), or β-glucuronidase (MPS VII) (2). The onset of MPS symptoms in humans varies between cases and subtypes but generally occurs in early childhood, and severely affected patients usually die during the second or third decade of life. Depending on the subtype, in addition to the CNS, the disease can also progressively affect the peripheral organs and/or skeleton.

Patients with neurological MPS experience a range of symptoms, including progressive cognitive impairment, sleep disturbance, and behavioral problems (1). Accumulation of HS within neurons and glia affects cell health, triggering secondary pathological cascades, including an autophagy blockage and accumulation of misfolded aggregated proteins such as α-synuclein, microtubule-associated protein tau, and amyloid-β (3–7). Other pathological cascades include synaptic defects, neuroinflammation, and astro- and microgliosis (7–11). In turn, induction of lysosomal biogenesis leads to the accumulation of lysosomes and the increase in the levels and activity of other lysosomal enzymes (4, 9, 12, 13).

In the present study, we report what we believe to be a novel pathological pathway manifesting in the neurological MPS spectrum. We show that in contrast to the majority of lysosomal enzymes, lysosomal neuraminidase 1 (NEU1), an enzyme involved in the cleavage of sialic acids from glycoproteins, shows a remarkable deficiency in the brain tissues of patients with neurological MPS and mouse models of the diseases. We further show that NEU1 deficiency in 2 models of MPS IIIC, KO, *Hgsnat-Geo* mouse (4) and the more severely affected knock-in *Hgsnat^P304L^* mouse (14) is caused by HS-mediated dissociation of its complex with other lysosomal enzymes—cathepsin A (CTSA), β-galactosidase (GLB1), and glucosamine-6-sulfate sulfatase (GALNS)—known as lysosomal multienzyme complex (LMC). Although NEU1 and the LMC are mainly known as lysosomal proteins, they also exist on the cell surface and act on cell surface glycoproteins (15). The results of the present study show that the secondary NEU1 deficiency in neurological MPS diseases leads to a widespread increase in sialylation of *N*-linked glycans and synaptic changes reminiscent of neuropathological findings that are both rescued by LV-mediated genetic correction of NEU1 activity in the MPS IIIC mouse brain.

## Results

### Secondary deficiency of NEU1 in tissues of patients with neurological MPS and mouse models.

**Figure 1:**
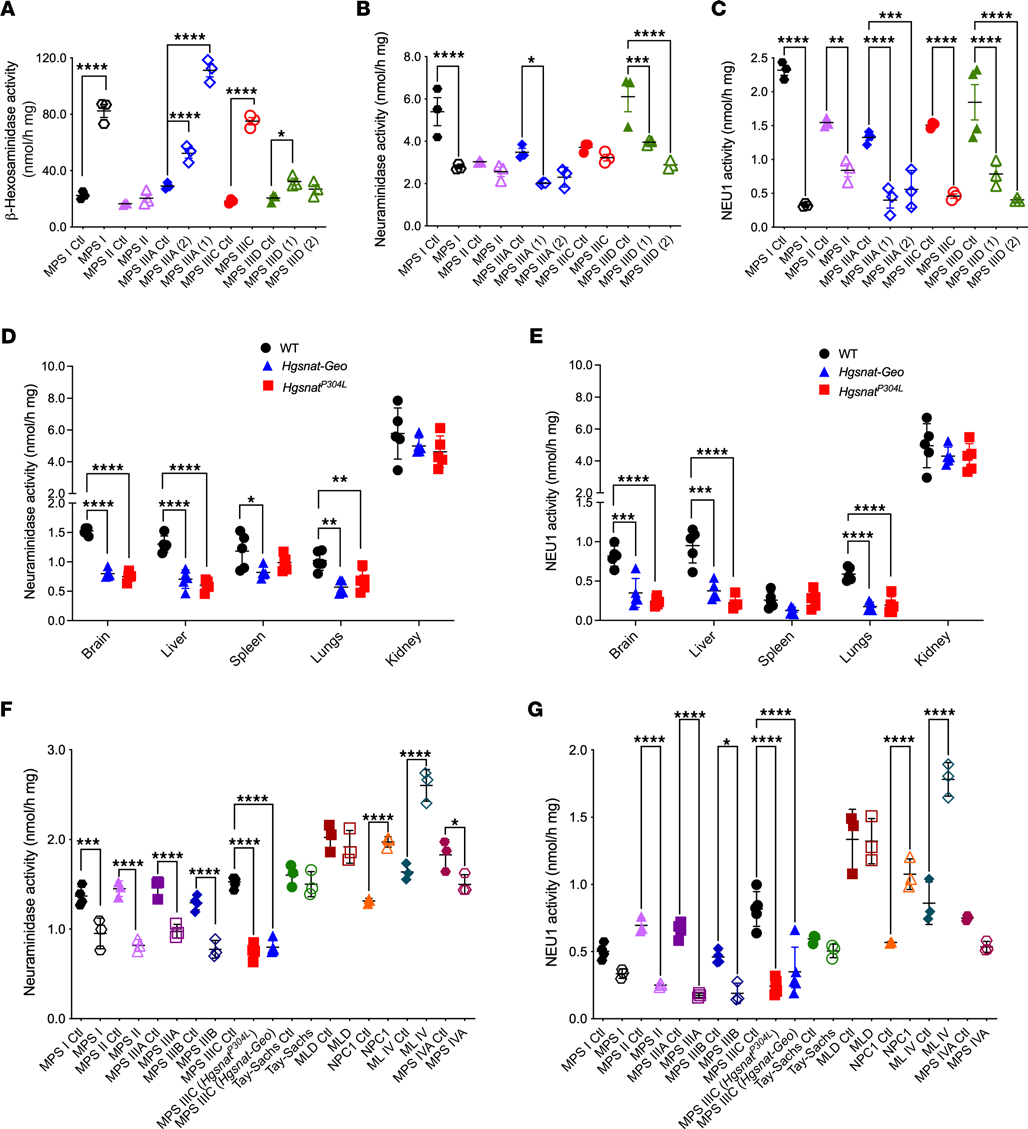
NEU1 activity is reduced in brain samples of patients with neurological MPS, brain tissues of neurological MPS mouse models and tissues of MPS IIIC mouse models. (**A**–**C**) Total β-hexosaminidase activity (**A**), total acidic neuraminidase activity (**B**), and NEU1 activity in the presence of a specific NEU3/4 inhibitor, C9-4BPT-DANA (**C**) in postmortem cortical tissues of patients with neurological MPS I, MPS II, MPS IIIA, MPS IIIC, and MPS IIID and corresponding age-, sex-, and ethnicity-matched control participants (Ctl; Supplemental Table 1). NEU1 activity is reduced in all samples from patients with MPS compared with those of control tissues. (**D**–**G**) Total neuraminidase (**D**) and NEU1 (**E**) activities are reduced in the brain, liver, and lungs of MPS IIIC mice compared with those of WT controls. Total neuraminidase (**F**) and NEU1 (**G**) activities are also reduced in the brains of the mouse models of neurological MPS disorders and MPS IVA compared with respective WT littermates, but they are similar to those of WT or are increased in other LSD models. Graphs show individual values and means (± SD) of 3–4 technical replicates (independent measurements of enzymatic activities in the same autopsy sample) per patient (**A**–**C**) or 3–5 mice (**D**–**G**). *P < 0.05, **P < 0.01, ***P < 0.001, ****P < 0.0001, determined by ANOVA with Šídák’s post hoc test (**A**–**C** and **F** and **G**) or Tukey’s post hoc test (**D** and **E**).

To characterize the biomarkers of CNS pathology in neurological MPS spectrum, we analyzed frozen somatosensory cortex postmortem tissues of 1 patient with MPS I, 1 with MPS II, 2 with MPS IIIA, one with MPS IIIC, 2 with MPS IIID, and of 7 control participants matched for age, sex, and ethnicity (project 1071, MPS Synapse, ref. 7; and Supplemental Table 1 for patient description; supplemental material available online with this article; https://doi.org/10.1172/JCI177430DS1). The combined activities of lysosomal β-hexosaminidases A and B were significantly increased in all patient samples except for MPS II, in which they showed a trend toward an increase (Figure 1A). This was consistent with the previously reported increase of lysosomal biogenesis and transcription factor EB–driven (TFEB-driven) expression of lysosomal genes in tissues and cells of patients affected with lysosomal storage diseases (4, 9, 12, 13). The changes were also reminiscent of the previously described increase of lysosomal enzyme activities and lysosome-associated membrane protein 2 levels in *Hgsnat^P304L^* and *Hgsnat-Geo* mouse models of MPS IIIC (4, 14). Unexpectedly, the total acidic neuraminidase activity, representing combined activities of NEU1, NEU3, and NEU4 isoenzymes, was either reduced or showed a trend toward a reduction (Figure 1B). Furthermore, the activity of NEU1 isoenzyme, measured in the presence of C9-4BPT-DANA, a potent inhibitor of both human and mouse NEU3 and NEU4 (IC_50_ 0.7 μM and 0.5 μM, respectively) with minimal activity against NEU1 (16), showed a drastic reduction in all brain tissues compared with their respective controls (Figure 1C). In contrast, NEU3/NEU4 activity deduced by subtraction of NEU1 from the total neuraminidase activity was unchanged (data not shown). This indicates that NEU1 is the neuraminidase affected in the MPS tissues. Similarly, total neuraminidase and NEU1 activities were drastically reduced in the brain, liver, and lungs, but not in the spleen and the kidney of both *Hgsnat^P304L^* and *Hgsnat-Geo* MPS IIIC mouse models compared with WT controls (Figure 1, D and E), whereas the total β-hexosaminidase and β-galactosidase activities were either increased or similar to those of controls (Supplemental Figure 1, A and B).

To test whether the secondary deficiency of NEU1 is shared by other lysosomal disorders, we analyzed total neuraminidase and NEU1 activities in the brain tissues of available MPS mouse models (MPS I, II, IIIA, IIIB, and IVA) and models of other neurological LSDs (namely, metachromatic leukodystrophy [MLD], Tay-Sachs, Niemann-Pick type C1 [NPC1], and mucolipidosis IV [ML IV]). All mouse models of neurological MPS I, II, IIIA, IIIB, and IIIC, characterized by HS accumulation, exhibited strongly reduced total neuraminidase and NEU1 activity, whereas activity levels in mouse models of other LSDs were unaffected (i.e., MLD, Tay-Sachs), or even increased (i.e., NPC1, ML IV) compared with their corresponding WT littermate controls (Figure 1, F and G). In the brains of the mouse model of MPS IVA, a non-neurological MPS, manifesting with some degree of HS storage in both patients and the mouse model (17, 18), the activity of NEU1 was decreased, but only by about 30%. This established NEU1 deficiency as a common phenomenon across all neurological MPS diseases in both mouse models and patients.

### Secondary deficiency of NEU1 is associated with lysosomal storage of HS.

**Figure 2:**
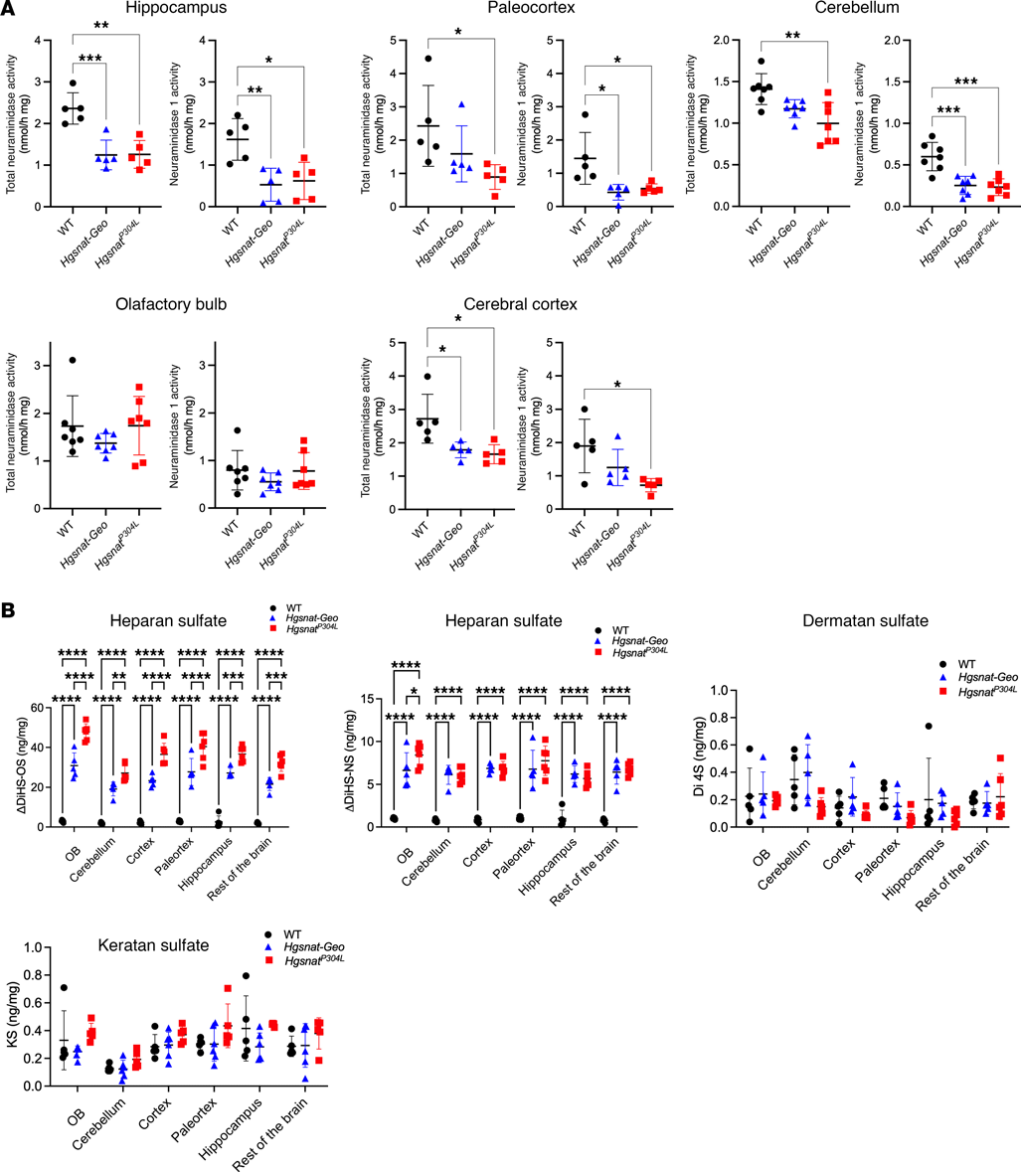
Secondary deficiency of NEU1 in the MPS IIIC mouse brain coincides with HS accumulation. (**A**) Total neuraminidase and NEU1 activity in the hippocampus, paleocortex, cerebellum, olfactory bulb (OB), and frontal cortex of 6-month-old WT, *Hgsnat-Geo,* and *Hgsnat^P304L^* mice. NEU1 activity is reduced in the hippocampus, paleocortex, cerebellum, and cerebral cortex. (**B**) Levels of disaccharides produced by enzymatic digestion of HS (ΔDiHS-OS and ΔDiHS-NS), dermatan sulfate (Di 4S), and monosulfated keratan sulfate (KS) were measured by MS/MS in dissected brain regions of WT, *Hgsnat^P304L^*, and *Hgsnat-Geo* mice. Individual data and means ± SD (*n* = 5–6) are shown. *P < 0.05, **P < 0.01, ***P < 0.001, ****P < 0.0001, determined by 1-way ANOVA with Tukey’s post hoc test (**A**) and 2-way ANOVA with Dunnett’s post hoc test (**B**).

X-Gal staining of brain sections from *Neu1* KO (*Neu1^–/–^*) mice expressing the bacterial LacZ/β-galactosidase reporter gene in the endogenous *Neu1* locus (19) identified the hippocampus, cerebellum, olfactory bulb, paleocortex, and deep layers of frontal cortex as the brain areas with the highest *Neu1* expression (Supplemental Figure 2). These regions were microdissected from brains of WT and MPS IIIC mice of both sexes and analyzed for total neuraminidase and NEU1 activities, which were found to be reduced in the hippocampus, paleocortex, frontal cortex, and cerebellum (Figure 2A).

**Figure 3:**
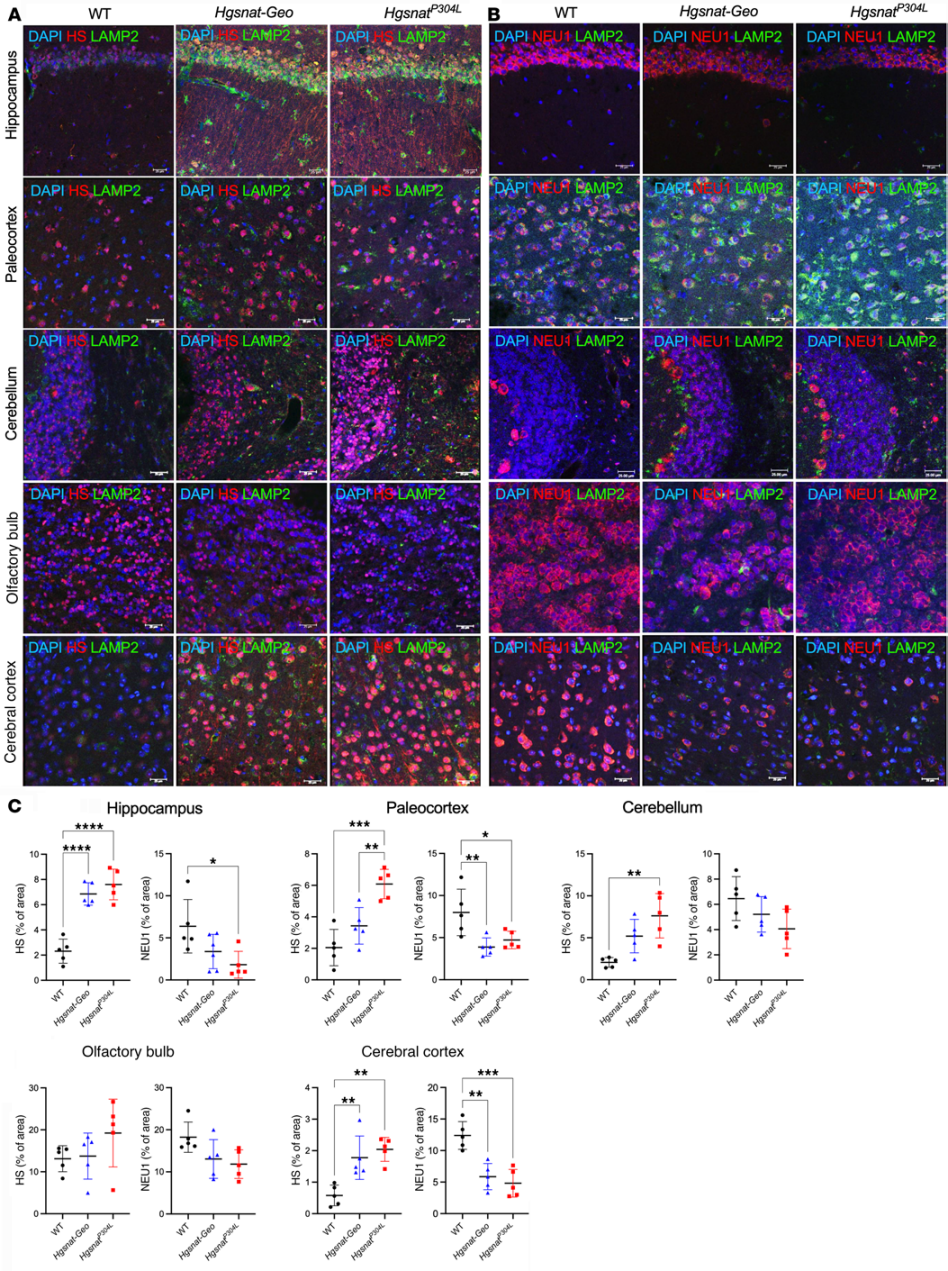
Reduced levels of NEU1 protein in the MPS IIIC mouse brain coincide with lysosomal storage of HS. Representative images (**A** and **B**) and quantifications (**C**) of HS and NEU1 immunoreactivity (red) of brain tissues of 6-month-old WT, *Hgsnat-Geo,* and *Hgsnat^P304L^* mice co-labeled with antibodies against lysosome-associated membrane protein 2 (LAMP-2; green), and DAPI (blue). HS is increased in the hippocampus, paleocortex, and frontal cortex of MPS IIIC mice compared with WT mice. *Hgsnat^P304L^* mice, but not *Hgsnat-Geo* mice, also show an accumulation of HS in the cerebellum. NEU1 is decreased in the hippocampus, paleocortex, and frontal cortex of MPS IIIC mice. Scale bars: 25 μm. Immunoreactivity was evaluated as percentage of total area using ImageJ software. Individual data and means ± SD (*n* = 5–7) are shown. *P < 0.05, **P < 0.01, ***P < 0.001, ****P < 0.0001, determined by nested 1-way ANOVA with Tukey’s post hoc test.

To test if the reduction of NEU1 activity is associated with HS accumulation and changes in NEU1 protein levels, brains of 6-month-old WT, *Hgsnat-Geo,* and *Hgsnat^P304L^* mice were analyzed by liquid chromatography–tandem mass spectrometry (LC-MS/MS) to assess levels of GAGs (Figure 2B) and by immunofluorescence with NEU1- and HS-specific antibodies (Figure 3). LC-MS/MS analysis confirmed increased levels of HS-derived O-sulfated (ΔDiHS-OS) and N-sulfated (ΔDiHS-NS) disaccharides in all brain areas of *Hgsnat-Geo* and *Hgsnat^P304L^* compared with WT mice, whereas the levels of GAGs, dermatan sulfate, and keratan sulfate were, as expected, not different from the controls (Figure 2B). It also revealed higher levels of ΔDiHS-OS in most of the brain areas of *Hgsnat^P304L^* mice compared with *Hgsnat-Geo* mice (Figure 2B). Levels of NEU1 were decreased and HS levels were drastically increased in the hippocampus, paleocortex, cerebral cortex, and cerebellum of *Hgsnat^P304L^* mice (Figure 3). Changes in *Hgsnat-Geo* mice were similar but less pronounced. Together, these results suggest the accumulation of HS in the brain is associated with deficiency of the NEU1 protein and activity.

**Figure 4:**
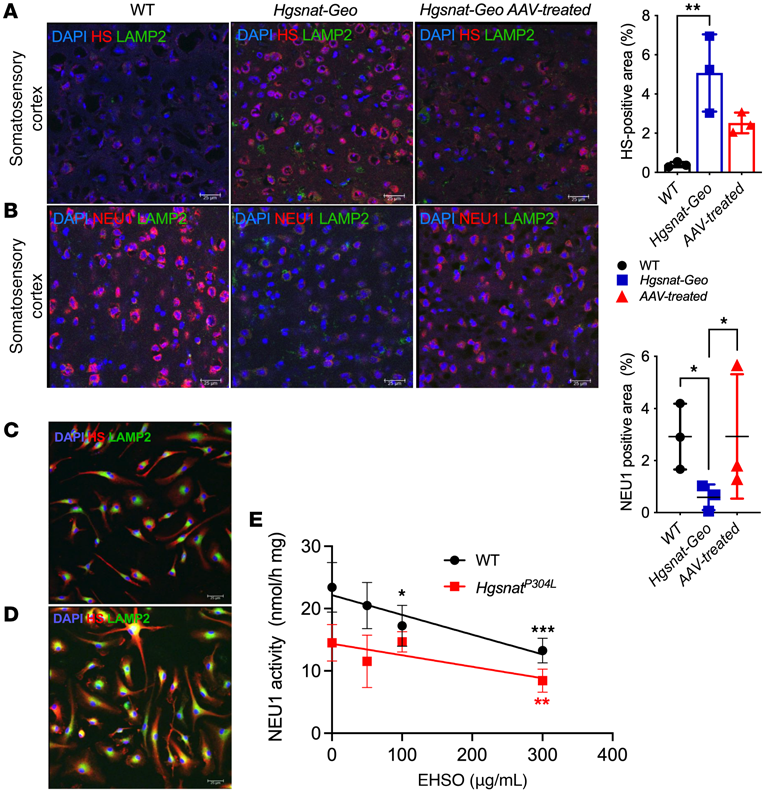
HS accumulation causes deficiency of NEU1. (**A** and **B**) HS storage is reduced and NEU1 levels increased in MPS IIIC mice treated with intracerebral injections of AAV-TT-HGSNAT vector. Brain sections of 6-month-old WT, untreated *Hgsnat-Geo*, and *Hgsnat-Geo* mice treated with AAV-TT-HGSNAT were labeled for (**A**) HS (red) and lysosome-associated membrane protein 2 (LAMP2; green) or (**B**) NEU1 (red) and LAMP2 (green). Nuclei were counterstained with DAPI (blue). HS shows a trend toward a decrease and NEU1 is increased in the brains of AAV-TT-HGSNAT–treated *Hgsnat-Geo* mice. Panels show representative images of the somatosensory cortex taken with a ×40 objective; scale bars: 25 μm. Data on the graphs show individual values and means ± SD (*n* = 3–5 areas per mouse). (**C**–**E**) Administration of EHSO to cultured BMDM causes secondary NEU1 deficiency. HS levels in cultured *Hgsnat^P304L^* BMDMs untreated (**C**) or treated with 300 μg/mL EHSO (**D**) were analyzed by immunofluorescence. Panels show representative images labeled for LAMP2 (green) and HS (red). Nuclei were counterstained with DAPI (blue). Scale bars: 25 μm. (**E**) NEU1 activity is progressively reduced in the BMDMs cultured in the presence of increasing concentrations of EHSO, as indicated. Confocal images were taken using a ×40 objective; scale bars: 25 μm. Data on the graphs show individual data and means ± SD (*n* = 4). *P < 0.05, **P < 0.01, ***P < 0.001, determined by nested 1-way ANOVA and a Tukey’s post hoc test (**A** and **B**), and 2-way ANOVA with a Dunnett’s post hoc test (**E**).

As described previously, i.c.v. injections of an adeno-associated virus (AAV) “true type” (AAV-TT) vector expressing human WT HGSNAT partially rescued HGSNAT deficiency in *Hgsnat-Geo* mice and reduced HS levels by approximately 46% (20). Concurrently, levels of neuroinflammation were reduced and behavioral deficits in the Y-maze and the open field (OF) tests were ameliorated (20). Therefore, we used the previously collected samples to test whether the treatment also rescued the secondary NEU1 deficiency. As shown by immunofluorescence, HS accumulation was reduced in *Hgsnat-Geo* mice with AAV-mediated HGSNAT expression, and this was paralleled by an almost complete restoration of NEU1 protein levels (Figure 4, A and B).

Cultured bone marrow–derived macrophages (BMDMs) from WT and *Hgsnat^P304L^* mice were used to determine whether reduced NEU1 activity was a consequence of HS accumulation in lysosomes. Extracellular HS oligomers (EHSOs), purified from the urine of patients with MPS IIIC, are readily taken up by cultured phagocytic cells such as macrophages or microglia (21). In our experiments, EHSO treatment increased HS in the lysosomal lumen (Figure 4, C and D), and reduced Neu1 activity of WT BMDMs in a dose-dependent manner (Figure 4E). Consistent with the results obtained with brain tissue samples (Figure 1B), NEU1 activity in untreated BMDMs from *Hgsnat^P304L^* mice was approximately 50% of the level in WT cells, but was also progressively reduced with increasing concentrations of EHSOs (Figure 4E). Notably, when EHSOs at similar concentrations were added to the homogenates of mouse brain tissues, we also observed a decrease of the NEU1 activity similar to that in the cultured cells (Supplemental Figure 3A). We did not observe, however, a reduction in the NEU1 activity in mouse kidney homogenates spiked with similar amounts of EHSOs, consistent with unchanged levels of the enzyme activity in this tissue (Figure S3B).

### HS induces secondary deficiency of NEU1 and disruption of the lysosomal multienzyme complex.

**Figure 5:**
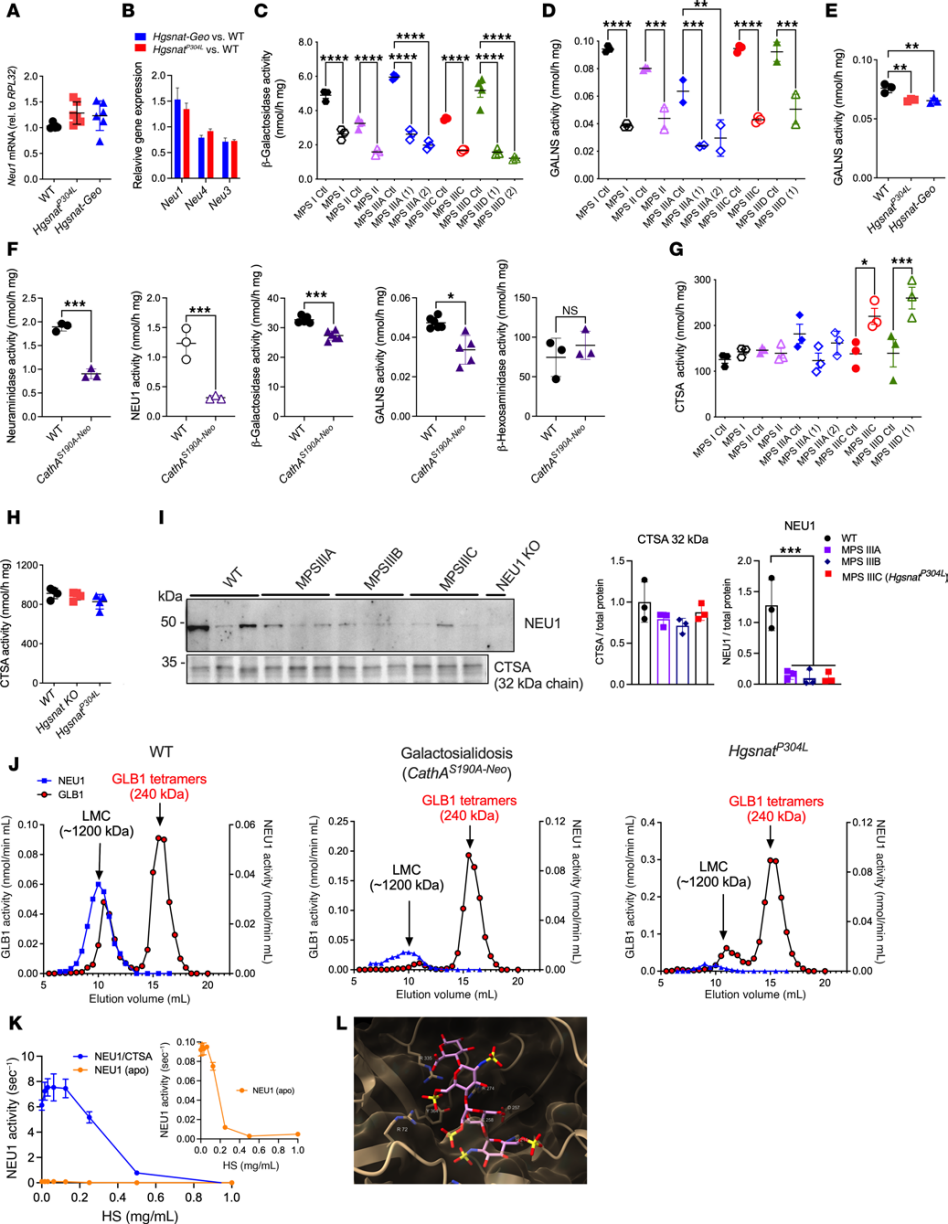
NEU1 deficiency in tissues and cells with lysosomal storage of HS is associated with the disruption of the LMC. (**A**) *Neu1* mRNA measured by quantitative RT-PCR and normalized for *Rpl32* expression. (**B**) *Neu1*, *Neu3*, and *Neu4* (relative to WT mice) expression measured in the hippocampi by total mRNA sequencing (14). (**C**) GLB1 activity is reduced in cortical tissues from patients with MPS I (561), MPS II (902), MPS IIIA (3617 and 563), MPS IIIC (6194), and MPS IIID (5411 and 5424)**,** compared with corresponding controls; numbers in parentheses refer to identity of neurological MPS patients in Supplemental Table 1. (**D**) GALNS activity is reduced in samples from patients with MPS compared with control samples. (**E**) GALNS activity is reduced in brains of MPS IIIC mice compared with WT mice. (**F**) NEU1, but not GLB1 or GALNS, is deficient in the brains of galactosialidosis *CathA^S190A-neo^* mice. (**G** and **H**) CTSA activity in brain samples of patients with MPS (**G**) and MPS IIIC mice (**H**) is similar or higher than that of controls. (**I**) Reduced NEU1 and unchanged CTSA protein levels in MPS IIIA-IIIC mouse brains compared with WT. (**J**) The gel-filtration profiles of total protein extracts from brain tissues of MPS IIIC *Hgsnat^P304L^*, WT, and *CathA^S190A-Neo^* mice and GLB1 and NEU1 activities in the eluted fractions. (**K**) HS inhibits NEU1 activity in vitro. Purified recombinant NEU1 was incubated with or without CTSA and HS, followed by NEU1 enzymatic activity (moles of substrate/moles of NEU1/sec) measurement. Insert shows expanded view of the results obtained with the NEU1 apo form. (**L**) Docked pose of an HS tetramer in the active site of NEU1 (8DU5). The arginine triad of NEU1 is able to engage sulfate and carboxylate groups of the ligand. All graphs show individual results and means ± SD; *n* = 3–5 (mice) or 3 independent measurements (human samples or recombinant enzymes). *P < 0.05, **P < 0.01, ***P < 0.001, ****P < 0.0001, determined by ANOVA with Tukey’s (**A**, **B**, **E**, and **H**) or Šídák’s (**C**, **D**, and **G**) post hoc tests or *t* test (**F**).

To test if HS induced the secondary deficiency of NEU1 by downregulating the *Neu1* gene expression, *Neu1* mRNA levels were measured by quantitative RT-PCR. However, comparable *Neu1* mRNA levels were detected in the brains of WT, *Hgsnat^P304L^*, and *Hgsnat-Geo* animals (Figure 5A). This result was also consistent with neuraminidase expression levels, derived from a previously published data set on gene expression in the hippocampus of MPS IIIC and WT mice (14) (Figure 5B). Together with the reduction of NEU1 protein in the brain of *Hgsnat^P304L^* and *Hgsnat-Geo* mice revealed by immunofluorescence analysis (Figure 3), these data suggest the NEU1 levels are altered at the post-transcriptional level.

NEU1, together with 3 other lysosomal enzymes (CTSA, GLB1, and GALNS), forms the LMC (22, 23). CTSA protects both GLB1 and NEU1 from lysosomal degradation and activates NEU1 (24), whereas LMC dissociation evoked by CTSA mutations leads to degradation of both NEU1 and GLB1 (25) and causes, in humans, the neurological LSD galactosialidosis.

Consistent with the assumption that the observed NEU1 deficiency in neurological MPS is caused by LMC dissociation, GLB1 and GALNS activities were reduced in all analyzed samples of patients with MPS to 25%-50% of the corresponding controls (Figure 5, C and D). Unlike the human enzymes, mouse GLB1 and GALNS appear to be stable and active in the absence of CTSA (26, 27). Indeed, in brain tissues of 4-month-old *Hgsnat^P304L^* and *Hgsnat-Geo* mice, only a small, although statistically significant, reduction in GALNS activity was observed (Figure 5E). This finding is reminiscent of the situation in *CathA^S190A-neo^* mice (the *Ctsa* hypomorph mouse model of galactosialidosis; ref. 27), in which NEU1 activity in the brain tissues was reduced to below 10% of normal, whereas GALNS and GLB1 activities were decreased by only approximately 30% and approximately 20%, respectively (Figure 5F).

In the brains of patients with MPS and of MPS IIIC mice, carboxypeptidase activity measured with the specific CTSA substrate CBZ-Phe-Leu was not reduced (Figure 5, G and H), ruling out that absence or deficiency of CTSA caused the LMC disruption and secondary NEU1 deficiency in these cases. Immunoblot analysis of brain homogenates confirmed unchanged CTSA protein levels in MPS IIIA, MPS IIIB, and MPS IIIC (*Hgsnat^P304L^*) mice (Figure 5I). In contrast, a drastic decrease in the NEU1 protein level was detected in all MPS III mice compared with WT mice (Figure 5I). The LMC integrity was further assessed by size-exclusion chromatography analysis of protein brain extracts (Figure 5J). The gel-filtration profiles of NEU1 and GLB1 activities were compared for the WT, *Hgsnat^P304L^*, and galactosialidosis *CathA^S190A-Neo^* mice. For the WT brain, both the intact approximately 1,200 kDa LMC and 240 kDa GLB1 tetramers (23) were detected by peaks of GLB1 enzymatic activity eluting from the column at retention times corresponding to approximately 1,200 kDa and 240 kDa, respectively. The majority of NEU1 activity was associated with the approximately 1,200 kDa LMC peak (Figure 5J). A major peak for the GLB1 tetramer, with no or small GLB1 and NEU1 activity peaks, corresponding to the elution volume of LMC, was detected in fractions from *CathA^S190A-Neo^* and *Hgsnat^P304L^* mice, respectively, indicating a dissociation of the LMC (Figure 5J).

### HS inhibits NEU1 activity and causes precipitation of CTSA, GBL1, and NEU1 proteins in vitro.

We further tested if HS would interfere with the activity of the recombinant purified NEU1 or NEU1-CTSA complex in vitro. The proteins were produced in Sf9 insect cells and purified as previously described (28, 29). Both hexahistidine-tagged and untagged CTSA and NEU1 proteins were tested to exclude the possibility of an unspecific interaction between HS and the positively charged tag. Our results demonstrated that high HS concentrations (≥0.3 mg/mL) reduced the activity of NEU1 in the complex with CTSA (Figure 5K) approximately 100-fold (i.e., to the levels of NEU1 not bound to CTSA). A similar effect was also observed for the unbound apo form of NEU1 (Figure 5K, insert). Moreover, incubation of CTSA, GLB1, and NEU1 at equimolar amounts with 1 mg/mL purified HS at acidic pH and in the presence of 100 mM NaCl, mimicking the conditions in the lysosomal lumen, caused precipitation of all 3 proteins, either isolated or in combination, suggesting that HS directly binds to each of them and interferes with the correct structural organization of their complex (Supplemental Figure 4A). In the absence of HS, all proteins remained soluble (Supplemental Figure 4B). Notably, hyaluronan, an anionic but not sulfated GAG, did not inhibit NEU1 or caused its precipitation even at the highest studied concentration of 1 mg/mL (data not shown).

Based on these findings, we conducted molecular docking of a HS tetramer to the NEU1 active site (AutoDock Vina, version 1.2.5) (30), which identified poses where the aminosulfate and carboxylate groups of HS could engage with the basic residues of the Arg triad in NEU1 and also gain additional contacts in the active site (Figure 5L). The pK_a_ of the iduronic acid carboxylate is known to be elevated relative to that of sialic acid (3–5 vs. 2.6) (31). Docking at the CTSA/GLB1 interface did not identify a distinct binding site, due to a shallower binding pocket. Further experimental support is required to confirm the specific interactions of HS with these proteins.

### Sialylation of N-linked glycans of brain glycoproteins but not polysialylation of NCAM is increased in patients with MPS and mice.

**Figure 6:**
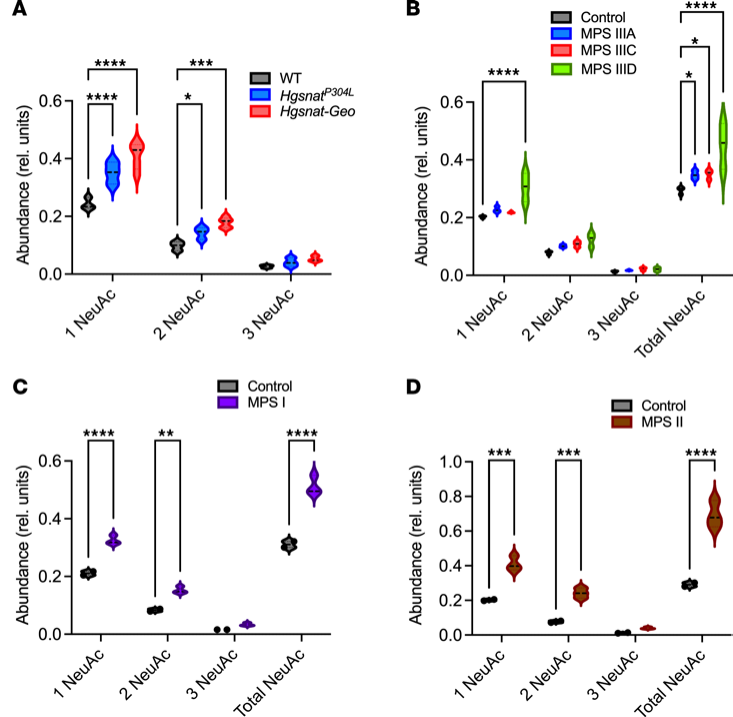
MALDI-TOF MS analysis shows increased sialylation of brain proteins in MPS mouse models and patients with MPS. Sialylation of N-linked glycans of brain glycoproteins from 10-month-old control, *Hgsnat^P304L^*, and *Hgsnat-Geo* mice (**A**); patients with MPS IIIA, IIIC, and IIID, as well as age- and sex-matched control participants (**B**); a patient with MPS I, as well as an age- and sex-matched control participant (**C**); and a patient with neurological MPS II, as well as an age- and sex-matched control participant (**D**) was analyzed by MALDI-TOF MS. All graphs show average relative intensity of MS peaks of glycan species containing 1, 2, or 3 NeuAc residues or of all sialylated glycans. *P < 0.05, **P < 0.01, ***P < 0.001, ****P < 0.0001, determined by 2-way ANOVA with Holm-Šídák’s multiple comparisons test. *n* = 3–5 (mice) or 1–3 patients per group. Rel., relative.

NEU1 normally cleaves sialic acid residues from glycans of glycoproteins either during lysosomal breakdown or at the cell surface (32). Therefore, to assess the effect of NEU1 deficiency on glycoprotein sialylation in MPS brains, *N*-linked brain glycans were analyzed by MALDI-TOF mass spectrometry. Compared with controls, multiple species of sialoglycans were elevated in both *Hgsnat^P304L^* and *Hgsnat-Geo* mice, including multiple isomeric species containing 2 or more sialic acid residues (Figure 6A and Supplemental Figure 5A). Similarly, highly sialylated glycans, particularly di-, tri-, and tetra-sialoglycans, were increased in the samples from patients with MPS IIIA, IIIC, and IIID (Figure 6B and Supplemental Figure 5B). In particular, a strong increase in a tetra-sialylated species at a mass-to-charge ratio (m/z) of 4761.3 (circled in red in Supplemental Figure 5B) was observed in MPS IIIC. MS/MS analysis of these species revealed fragments consistent with at least 4 distinct isomeric structures differing in the position of terminal sialic acid and fucose units (data not shown). In MPS I and II, a general increase in sialylated species was detected together with a widespread increase in complex fucosylated species compared with oligomannose structures (Figure 6, C and D, and Supplemental Figure 5, C and D). In MPS I, MS/MS analysis of the increased sialoglycans at m/z 4213.1 and 4574.3 revealed an enhanced content of glycoisomers bearing antennary sialyl-Lewis epitopes (circled in red in Supplemental Figure 5C) uncommon in the human brain. In MPS II, 2 minor unfucosylated glycoforms, at m/z 2431.2 and 2792.3 (circled in red in Supplemental Figure 5D) with 1 or 2 antennas each bearing a sialic acid residue, were markedly increased. Taken together, these data indicate that the secondary deficiency of NEU1 leads to oversialylation of brain glycans in all studied cases of neurological MPS.

Because NEU1 is highly active toward sialylated glycoproteins (15), patients with sialidosis with primary NEU1 deficiency have lysosomal accumulation and massive urinary excretion of sialic acid–containing oligosaccharides. To test whether this also occurs in the patients with MPS III, we analyzed by MALDI-TOF MS soluble glycans in the urine of 3 pediatric patients with MPS IIIC and 3 healthy, age-matched control participants. We found that the urine of patients with MPS IIIC contained multiple undegraded bi- tri-, and tetrantennary sialoglycans with a single *N*-acetylglucosamine residue at the reducing end (Supplemental Figure 6A) resembling those found in the urine of patients with sialidosis and galactosialidosis (33). Such structures were absent in the samples from healthy control participants (Supplemental Figure 6B), confirming that the secondary NEU1 deficiency in the patients with MPS IIIC results in the inadequate catabolism of sialylated oligosaccharides. Notably, the presence of sialylated oligosaccharides also recently has been reported in the urine of 2 patients with MPS IIIA (34).

**Figure 7:**
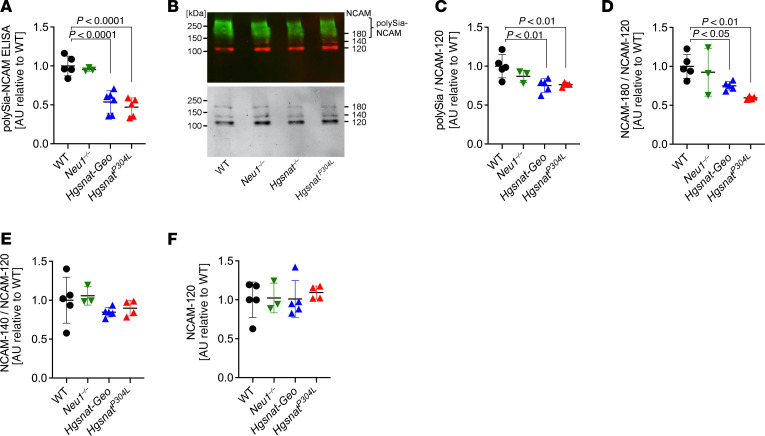
ELISA and Western blot analyses show reduced polySia-NCAM in the cortex of MPS IIIC mouse models. (**A**) PolySia-NCAM levels in lysates of the anterior cortex from 4-month-old WT, *Neu1^–/–^*, *Hgsnat^—^Geo*, and *Hgsnat^P304L^* mice were assessed by sandwich ELISA. Values were normalized to the mean values of the WT controls. For each sample from 1 animal, 4 technical replicates were measured in independent experiments, and individual values with means (± SD) of 3–6 animals per genotype are plotted. (**B**) Example of Western blot detection with polySia- (800 nm, green, upper panel) and NCAM-specific antibodies (700 nm, red, upper panel) and grayscale image (lower panel). (**C**–**F**) Densitometric evaluation of polySia-NCAM Western blot signals (**C**) and the bands characteristic for the polySia-free NCAM-140 and NCAM-180 isoforms (**D** and **E**), normalized to the mean values of the WT controls. Values were normalized to the intensities of NCAM-120, the isoform of mature oligodendrocytes, which is not subject to polysialylation in the mouse brain (35). (**F**). Individual values with means (± SD) of 3–6 animals per genotype are plotted.

In contrast to the hypersialylation of other *N*-linked glycans, a drastic reduction in the polysialylated (polySia) NCAM was detected in samples from the anterior cortex of both *Hgsnat^P304L^* and *Hgsnat-Geo* mice, but not *Neu1^–/–^* mice, when measured by a polySia-NCAM–specific sandwich ELISA (Figure 7A). Analysis of the same set of samples by Western blot with polySia-specific antibodies revealed a reduction of the high-molecular-weight smear bands, characteristic of the polySia NCAM isoforms (35, 36). The NCAM-180 protein band detected with NCAM-specific antibodies was also drastically reduced; NCAM-140 and NCAM-120 protein bands remained at the WT levels (Figure 7, B–F). Together, these findings demonstrate that polySia, unlike mono, di-, and oligo-sialylated *N*-glycans, are not increased by reduced NEU1 activity.

### Overexpression of NEU1 and CTSA in human iPSC–derived cultured cortical MPS IIIA neurons partially rescues synaptic defects.

**Figure 8:**
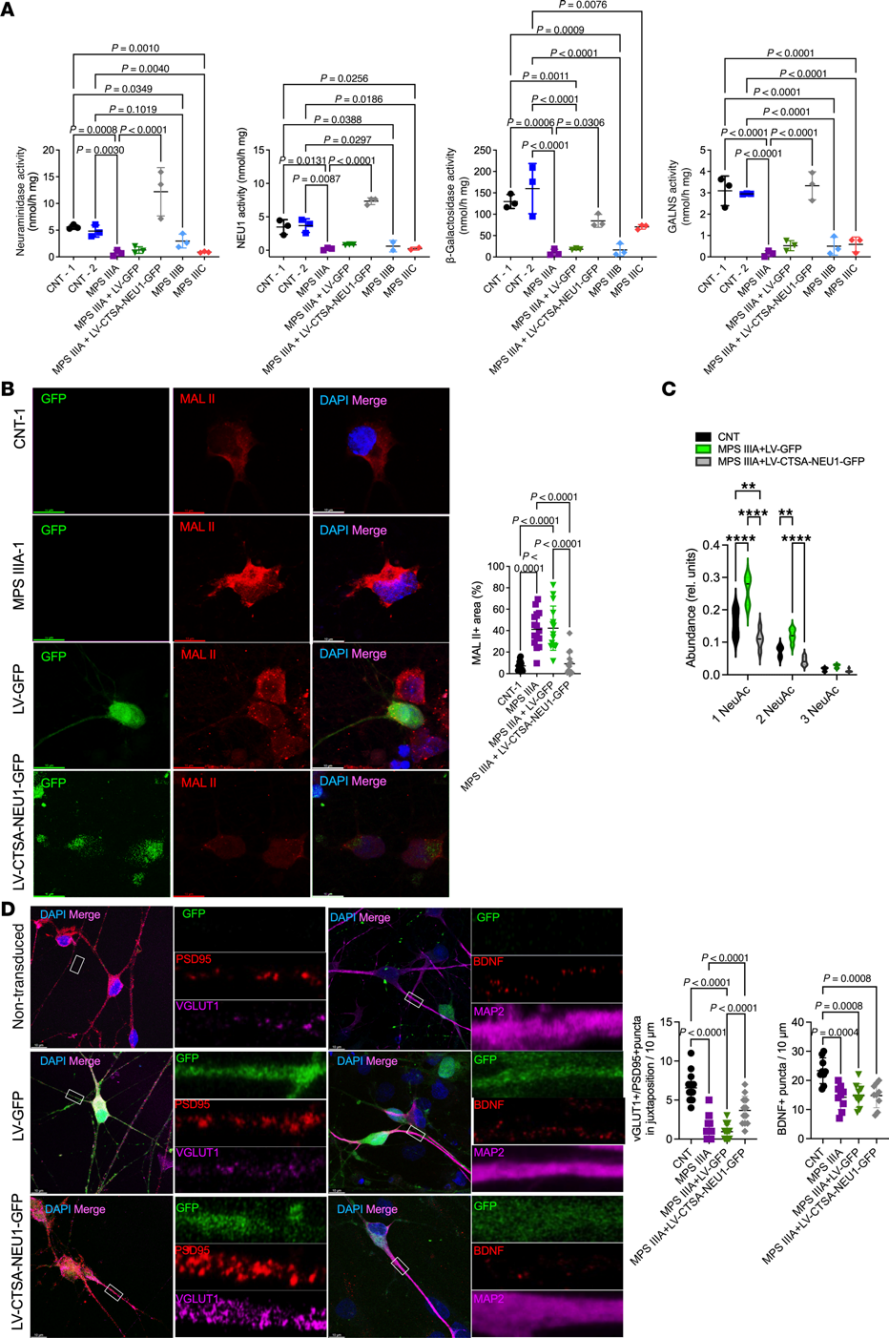
Overexpression of NEU1 and CTSA rescues deficient NEU1, GLB1, and GALNS activities and reduced levels of glutamatergic synapses in human cortical iPSC-derived MPS IIIA neurons. (**A**) NEU1, GLB1, and GALNS activities are markedly reduced in cultured iPSC-derived cortical neurons of patients with MPS IIIA, IIIB, and IIIC compared with cells from 2 healthy control participants (CNT-1 and CNT-2). Normal levels of NEU1, GLB1, and GALNS activities are restored in MPS IIIA cells transduced with LV-CTSA-IRES-NEU1-GFP but not with LV-GFP. The graphs show individual data and means ± SD (*n* = 3), with significance determined by ANOVA with Dunnett’s post hoc test. (**B**) MPS IIIA iPSC–derived neurons show increased labeling with MAL II lectin compared with control cells, consistent with increased surface sialylation. The labeling is reduced in the MPS IIIA cells transduced with LV-CTSA-IRES-NEU1-GFP but not with LV-GFP. (**C**) Sialylation of *N*-linked glycans of cultured neurons analyzed by MALDI-TOF MS is increased in MPS IIIA cells transduced with LV-GFP and decreased in cells transduced with LV-CTSA-IRES-NEU1-GFP. Graphs show average relative intensity of MS peaks of glycan species (Supplemental Table 2) containing 1, 2, or 3 NeuAc residues. *n* = 5. *P* values were calculated by 2-way ANOVA with Holm-Šídák’s multiple comparisons test. (**D**) MPS IIIA iPSC–derived neurons have lower densities of PSD-95^+^ puncta in juxtaposition with VGLUT1^+^ puncta compared with control cells, revealing the decrease in the number of functional synapses. The density is increased in LV-CTSA-IRES-NEU1-GFP–transduced but not in LV-GFP–transduced MPS IIIA cells. The amount of BDNF^+^ puncta (red) is reduced in nontransduced MPS IIIA neurons or those transduced with LV-GFP and LV-CTSA-IRES-NEU1-GFP compared with the control. Scale bars: 10 μm. Fluorescent areas were quantified by ImageJ software. Puncta were counted manually along the axon at 30 μm increments starting 10 μm from the soma and averaged to 10 μm. Graphs show individual data (*n* = 9–15) and means ± SD, with significance determined by 1-way ANOVA and a Dunnett’s post hoc test.

To test whether reduction of NEU1 and increased sialylation of brain glycoproteins contribute to synaptic defects in patients with MPS III and mouse models (14, 37–39), we analyzed periaxonal PSD-95^+^ puncta in juxtaposition with VGLUT1^+^ puncta in cortical iPSC-derived neurons. NEU1, GALNS, and GLB1 activities were drastically reduced in MPS IIIA, MPS IIIB, and MPS IIIC neurons compared with healthy controls (Figure 8A). All activities were restored in the MPS IIIA neurons transduced with the previously described LV bicistronic vector LV-CTSA-IRES-NEU1-GFP and overexpressing human GFP-tagged-NEU1 and CTSA (overexpression of the NEU1 without CTSA results in its aggregation in the cytoplasm) (40). LV transduction of MPS IIIA neurons with LV-CTSA-IRES-NEU1-GFP, but not with control LV-GFP virus, also reduced the affinity of cells to MAL II lectin, specific for glycans with terminal 2,3-linked sialic acid residues (Figure 8B). Fluorescence lectin labeling results were confirmed by the results of MALDI-TOF MS analysis demonstrating that sialylation of *N*-linked glycans of cultured neurons (Supplemental Table 2) was increased in MPS IIIA cells transduced with LV-GFP and decreased in cells transduced with LV-CTSA-IRES-NEU1-GFP (Figure 8C). Notably, the cells transduced with LV-CTSA-IRES-NEU1-GFP but not with LV-GFP showed a drastic increase in densities of juxtapositioned PSD-95^+^ and VGLUT1^+^ puncta to the level statistically similar to that in control cells (Figure 8D). Because NEU1 was reported to induce the release of brain-derived neurotrophic factor (BDNF) (41), BDNF levels in the neurons were assessed by immunofluorescence. BDNF levels in MPS IIIA neurons were below control levels and not affected by transduction with LV-CTSA-IRES-NEU1-GFP or LV-GFP (Figure 8D).

### Stereotaxic injection of LV-CTSA-IRES-NEU1-GFP in the brain of MPS IIIC mice ameliorates behavior abnormalities and CNS pathology.

To test whether a rescue of NEU1 activity in the brain could also ameliorate known behavioral deficits of MPS IIIC mice (4, 14), we performed a bilateral injection of LV-CTSA-IRES-NEU1-GFP in the hippocampi and cortices of P18-P19 *Hgsnat^P304L^* mice. Based on pilot experiments, we determined injection coordinates as 1.5 mm mediallateral, 2.2 mm anteroposterior, 2.2 mm dorsoventral, and 1.2 mm dorsoventral to be optimal for targeting the CA1 area of the hippocampus and the layers IV and V of the somatosensory cortex.

**Figure 9:**
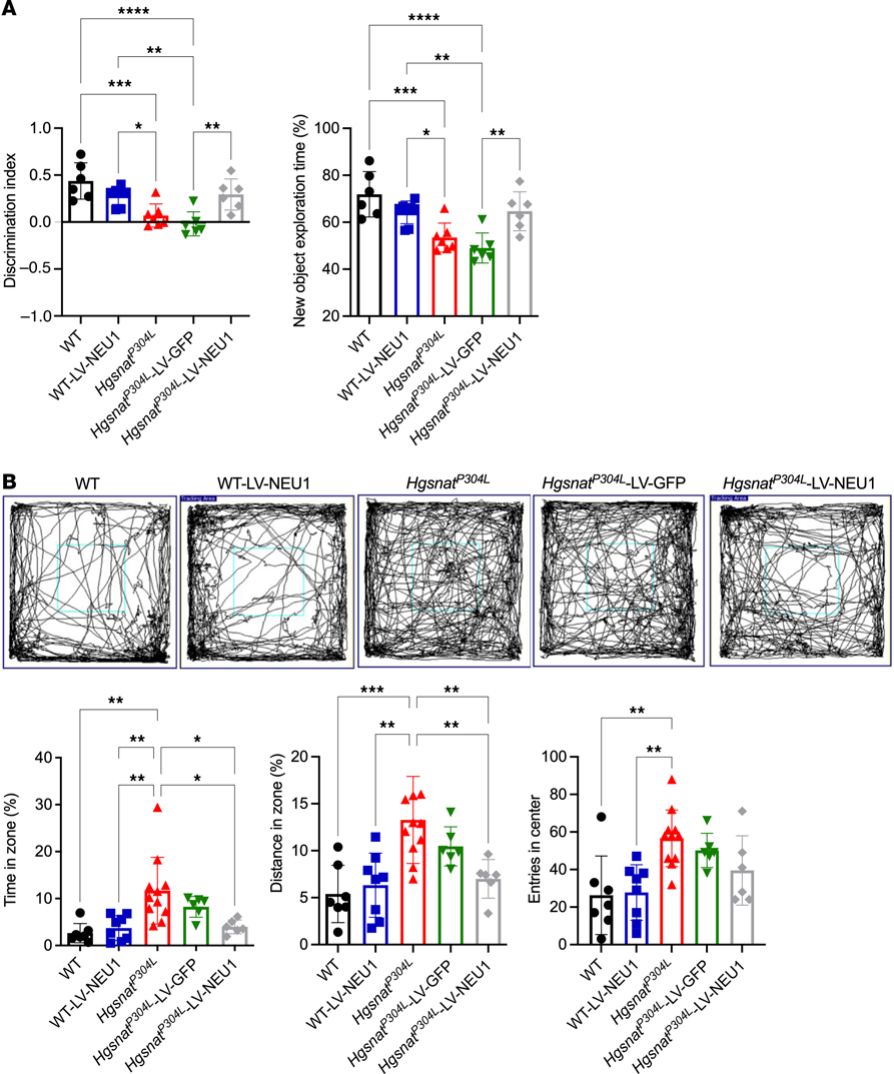
*Hgsnat^P304L^* mice treated with LV-CTSA-IRES-NEU1-GFP show a partial rescue of behavior abnormalities. (**A**) Improvement of the short-term memory in *Hgsnat^P304L^* mice injected with LV-CTSA-IRES-NEU1-GFP. Discrimination index and the percentage of time spent exploring the novel object were measured using the NOR test in 6-month-old WT mice, WT mice injected with LV-CTSA-IRES-NEU1-GFP, *Hgsnat^P304L^* mice, and *Hgsnat^P304L^* mice injected with LV-CTSA-IRES-NEU1-GFP or LV-GFP. *Hgsnat^P304L^* mice injected with LV-CTSA-IRES-NEU1-GFP had a higher discrimination index and more time spent with the new object than did control or sham-treated *Hgsnat^P304L^* mice. (**B**) *Hgsnat^P304L^* mice injected with LV-CTSA-IRES-NEU1-GFP show normalization of anxiety as revealed by the OF test. Graphs show the percentage of time spent in the center of the arena, the percentage of the distance traveled in the center zone, and the number of entries to the center of the arena. All graphs show individual data and means ± SD (*n* = 6–11). **P < 0.01, ***P < 0.001, ****P < 0.0001, determined by 1-way ANOVA with a Tukey’s post hoc test.

Six months after the treatment, we analyzed the behavior of LV-CTSA-IRES-NEU1-GFP–injected, sham LV-GFP–injected and control WT and *Hgsnat^P304L^* mice using a novel object recognition (NOR) test to assess short-term memory and an OF test to study activity and anxiety levels, as we previously reported (14). In NOR test with an interval of 1 hour between encoding and retrieval, both treated and untreated WT mice showed a positive discrimination index, indicating they spent more time exploring a novel object in the retrieval phase—compatible with uncompromised short-term recognition memory (Figure 9A). For untreated or sham-treated *Hgsnat^P304L^* mice, the discrimination index was close to zero, and the time spent with the novel object was reduced. However, for *Hgsnat^P304L^* mice injected with LV-CTSA-IRES-NEU1-GFP, both parameters were similar to those of WT mice, suggesting a rescue of the short-term memory deficit (Figure 9A).

In the OF test, untreated *Hgsnat^P304L^* mice of both sexes spent significantly more time and traveled longer distances in the center zone compared with untreated or treated WT mice (Figure 8B). In contrast, time spent and distance traveled in the center zone for both male and female *Hgsnat^P304L^* mice injected with LV-CTSA-IRES-NEU1-GFP (but not with LV-GFP) was reduced to WT levels consistent with normalization of anxiety (Figure 9B).

Histological analysis of mice following the behavioral tests revealed a widespread expression of both GFP and NEU1-GFP proteins in the neurons of hippocampus, especially the granule cells of the dentate gyrus, the hilar mossy cells, and the CA2/CA3 areas (Supplemental Figure 7), but not in the neurons of somatosensory cortex (SSC). Such a distribution was not surprising considering that the LV infects mainly dividing or young cells, which are found in the dentate gyrus of the hippocampal formation, but not in the SSC (42).

**Figure 10:**
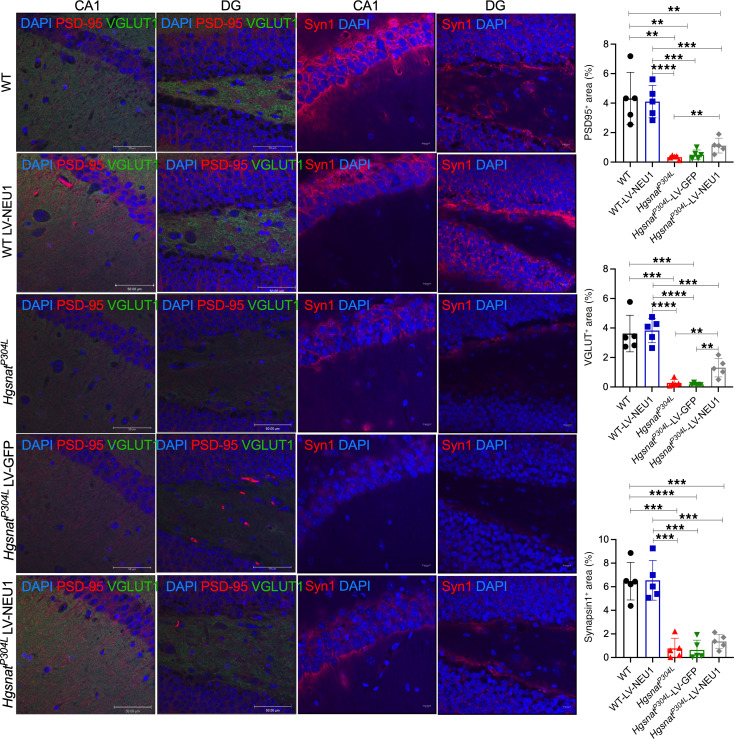
Levels of protein markers of glutamatergic synapse are increased in the brains of *Hgsnat^P304L^* mice injected with LV-CTSA-IRES-NEU1-GFP. Representative confocal images and quantification of fluorescence in 4 regions of the hippocampus of WT mice, WT mice injected with LV-CTSA-IRES-NEU1-GFP (LV-NEU1), *Hgsnat^P304L^* mice, *Hgsnat^P304L^* mice injected with LV-GFP, and *Hgsnat^P304L^* mice injected with LV-CTSA-IRES-NEU1-GFP. The sections were labeled for PSD-95 (red) and VGLUT1 (far red/pseudo green) or for Syn1 (red), as indicated. The nuclei were counterstained with DAPI (blue). Quantification of images demonstrates that LV-CTSA-IRES-NEU1-GFP treatment increases the levels of both PSD-95 and VGLUT1 in *Hgsnat^P304L^* mice, whereas Syn1 shows a nonsignificant trend for an increase. Scale bars: 50 and 10 μm. Data on the graphs show individual results and means (± SD) (*n* = 5). **P < 0.01, ***P < 0.001, ****P < 0.0001, determined by 1-way ANOVA with Tukey’s post hoc test.

To test for a rescue of synaptic defects, we analyzed VGLUT1, PSD-95, and Syn1. In agreement with previous findings (14), levels of VGLUT1, PSD-95, and Syn1 puncta in the hippocampi of *Hgsnat^P304L^* and the sham-treated *Hgsnat^P304L^* mice were significantly decreased compared with those of WT mice. Injection of LV-CTSA-IRES-NEU1-GFP did not alter levels of the synaptic proteins in WT mice but led to a significant increase of VGLUT1 and PSD-95 levels in *Hgsnat^P304L^* mice, whereas the levels of Syn1 showed a nonsignificant trend toward an increase (Figure 10). Consistent with the findings for whole-brain extracts (Figure 6A), MALDI-TOF MS and MS/MS analysis indicated increased sialylation of *N*-linked glycans in the hippocampus of untreated *Hgsnat^P304L^* mice compared with WT mice, which was normalized by LV-CTSA-IRES-NEU1-GFP injection (Supplemental Figure 8A). Notably, MS/MS analysis revealed that only levels of 2,3- or 2,6-linked sialic acids were increased in untreated *Hgsnat^P304L^* mice and decreased in LV-CTSA-IRES-NEU1-GFP–injected *Hgsnat^P304L^* mice, suggesting that NEU1 was active against these types of linkage (Supplemental Figure 8B). In contrast, glycans bearing 2,8-linked sialic acids were either unaffected or showed a trend opposite to those containing 2,3- or 2,6-linkages. This is consistent with unaltered levels of 2,8-linked polySia in *Neu1^–/–^* mice (Figure 7).

Amyloid deposits are among the hallmarks of MPS III (4, 5, 7, 43) and are associated with increased amyloid β secretion in *Neu1* KO mice (44). Consistent with these findings, β-amyloid protein levels were increased in *Hgsnat^P304L^* mice but were unaffected by LV-CTSA-IRES-NEU1-GFP injection (Supplemental Figure 9). We also found no changes in the levels of GM2 ganglioside, 1 of the main secondary storage materials in the brains of patients and mice with MPS III (Supplemental Figure 10).

## Discussion

The present study reveals a likely previously unknown pathophysiological pathway in neurological MPS diseases caused by accumulation of HS, manifesting with a secondary deficiency of NEU1 and leading to synaptic defects. While studying the secondary changes in the activities of lysosomal enzymes in the brain tissues of *Hgsnat^P304L^* and *Hgsnat-Geo* MPS IIIC mouse strains, we serendipitously discovered an approximately 50% reduction in total neuraminidase activity in both models. A reduction of neuraminidase activity was also reported for the brain tissues of MPS IIID goats and MPS IIIB mice but remained unexplained (13, 45). An even more drastic decrease was observed in the activity of the lysosomal NEU1 isoform, measured in the presence of the specific inhibitor of NEU3 and NEU4, C9-4BPT-DANA (16). Because increased lysosomal biogenesis, linked to activation of TFEB-driven expression of lysosomal genes (4, 9, 12, 13), occurs in multiple mouse models of neurological MPS, including those of MPS IIIC (4, 9, 12, 14, 46–50), NEU1 activity showed a trend opposite to that of other lysosomal enzymes and proteins.

We further found a correlation between the levels of HS storage and the reduction of NEU1 activity and protein levels in different brain regions of MPS IIIC mice. In particular, NEU1 was deficient in the hippocampus and SSC, the areas with high storage of HS, but not in the cerebellum, where HS accumulated at a lower level. Likewise, the pronounced approximately 80% decrease of NEU1 activity in liver and lungs coincided with high levels of HS. In contrast, the kidney, an organ with high NEU1 levels (~10-fold higher than that in the brain) but relatively low HS storage (12), showed no NEU1 deficiency. Alternatively, the kidney enzyme (or LMC) could possess a different property that protects it from inhibition by HS. Further studies are necessary to test this hypothesis, but it seems consistent with a low sensitivity of NEU1 to EHSO in mouse kidney compared with brain homogenates.

The secondary NEU1 deficiency occurred in the brains of all analyzed mouse models of neurological MPS (MPS I, II, IIIA, IIIB, IIIC) but not in the models of other LSDs (metachromatic leukodystrophy, ML IV, Tay-Sachs, or NPC1). This suggests that NEU1 deficiency is shared only by the lysosomal disorders that store HS. MPS IVA mice, which accumulate mainly keratan sulphate, seem to defy this trend, because they showed a slight but statistically significant reduction in the brain NEU1 activity. However, some patients with MPS IVA also have increased tissue levels of HS (51). Thus, a respective HS storage may occur in the brain of MPS IVA mice and contribute to the partial NEU1 deficiency. Most importantly, our data show that NEU1 deficiency manifests in the brains of patients with neurological MPS, making this finding relevant to our understanding of human pathology.

When BMDMs, known to internalize exogenous HS into the lysosomes (52), were treated with urinary HS oligomers resembling those accumulated in the brain (53), NEU1 activity was reduced proportionally to the HS levels in the culture medium. Reciprocally, AAV-mediated genetic correction of HGSNAT deficiency and HS storage in the brain of *Hgsnat-Geo* mice restored NEU1 activity, proving that NEU1 deficiency was caused by the primary storage of HS and establishing a causal relationship between HS storage and NEU1 deficiency.

The amount of *Neu1* mRNA in the brain tissues of *Hgsnat^P304L^* and *Hgsnat-Geo* mice was similar to WT, whereas the amount of NEU1 protein in neurons was reduced, suggesting that the mechanism of the deficiency was post-translational, possibly associated with reduced NEU1 stability. A similar phenomenon occurs in the cells of patients with galactosialidosis with a genetic deficiency of CTSA leading to disruption of the LMC (23, 25). Notably, the activities of 2 other LMC components, GLB1 and GALNS, stabilized by their association with CTSA (23), were reduced by at least 50% in brain tissues of patients with MPS and cultured iPSC-derived MPS IIIA neurons; in *Hgsnat^P304L^* mice brain extracts, however, GLB1 was present in the form of homotetramers instead of LMC. Together, these results implicate HS in the dissociation of the complex leading to secondary NEU1, GLB1, and GALNS deficiencies in the brains of patients with neurological MPS. In contrast, in MPS IIIC mouse brains, NEU1 was deficient but GLB1 was increased, and GALNS was only slightly reduced, as in the tissues of galactosialidosis *CathA^S190A-neo^* mice, consistent with high stability of mouse GLB1 and GALNS enzymes in the lysosome (27). These in vivo results were partially corroborated by in vitro analysis demonstrating that high HS concentrations caused precipitation of human purified recombinant NEU1, GLB1, and CTSA and the complete loss of NEU1 activity. Computational prediction of specific interactions between HS and proteins can be challenging (54–56). Based on the data presented here, we hypothesize that HS interacts either with NEU1 or the CTSA/GLB1 protein-protein interface disrupting the correct organization of the LMC and making NEU1 and GLB1 enzymes more susceptible to lysosomal degradation. However, more studies are necessary to identify the underlying structural mechanism and clarify whether storage of HS also causes deficiency of other lysosomal enzymes or proteins.

The level of protein sialylation in the brain is not static; it changes dramatically during development and in response to physiological conditions. Moreover, neuraminidases, including NEU1, are at least partially responsible for these changes (15). In particular, intracerebral injections of the pan-neuraminidase inhibitor DANA resulted in a drastic decrease in LTP, an increase in short-term depression, and an alteration of synaptic plasticity in rodents (57). Similarly, DANA inhibited long-term potentiation at mossy fiber-CA3 synapses of rats and increased their escape latency in the Morris water maze test (58). The same group reported that NEU activity was increased after neuronal excitation and that the removal of sialic acid from the neuronal surface was critical for hippocampal memory formation in a contextual fear-conditioning paradigm (59). In this study, MS analysis of *N*-linked glycans revealed that NEU1 deficiency in *Hgsnat^P304L^* and *Hgsnat-Geo* mice, as well as in patients with MPS I, II, and III, was associated with an overall increase in the sialylation of brain glycoproteins, which could potentially modify hippocampal synaptic transmission and plasticity.

Consistently, *Hgsnat^P304L^* mice injected with the LV-CTSA-IRES-NEU1-GFP had higher levels of both pre- and postsynaptic glutamatergic protein markers VGLUT1 and PSD-95, respectively, in the hippocampus, consistent with amelioration of glutamatergic synaptic defects. The number of Syn1^+^ puncta also showed a trend toward an increase in the cells overexpressing NEU1. Similar results were observed in human iPSC–derived cultured MPS IIIA neurons transduced with the LV-CTSA-IRES-NEU1-GFP. Consistent with an increase in the levels of glutamatergic synaptic proteins, we also observed an improvement in short-term memory and partial rescue of reduced anxiety in *Hgsnat^P304L^* mice treated with LV-CTSA-IRES-NEU1-GFP. This partial improvement of behavior could be directly linked to the increased NEU1 activity and amelioration in glutamatergic neurotransmission in the hippocampus, the brain area responsible for short-term memory and modulation of anxiety.

Deficiency of NEU1 in the brain has been previously implicated in amyloidogenesis (44). *Neu1* KO mice spontaneously developed amyloid plaques, presumably due to increased release of hypersialylated amyloid precursor protein. In contrast, plaques were markedly decreased in a mouse model of Alzheimer’s disease treated with AAV9 overexpressing NEU1 (44). Because amyloidogenesis also occurs in MPS III brain, it could be related to NEU1 deficiency. Our experiments, however, could neither confirm nor reject the link between the NEU1 deficiency and amyloidogenesis in the MPS III. In MPS IIIC mice, accumulation of misfolded amyloid protein occurs mainly in the level 4 and 5 pyramidal neurons of the cortex (59), where we could not observe any expression of the NEU1-GFP after the injection of the LV. Thus, although β-amyloid staining in the neurons of untreated *Hgsnat^P304L^* mice was similar to those treated with LV-CTSA-IRES-NEU1-GFP, we cannot exclude that the absence of the effect was due to the lack of NEU1 expression. In contrast, the hippocampal neurons that showed high transduction with LV-CTSA-IRES-NEU1-GFP do not, in general, reveal any β-amyloid accumulation in MPS IIIC. Therefore, further experiments, involving targeted transduction of cortical neurons with the NEU1-expressing virus, should be performed to test the association of NEU1 deficiency with amyloidogenesis. Notably, we also could not detect any reduction in the secondary storage of GM2 ganglioside in the cells overexpressing NEU1, which suggested that the NEU1 deficiency does not contribute to the impairment of ganglioside catabolism and/or autophagy in MPS IIIC neurons.

It has been proposed that NEU1 plays a role in degradation of polySia attached to NCAM and perhaps to other brain proteins. The data from the present study, however, did not reveal an increase in polysialylation of NCAM, the major polySia-modified protein in the brain. In contrast, in the brain of the 2 MPS IIIC mouse models with secondary Neu1 deficiency, we observed a marked reduction in polySia-NCAM and of the NCAM-180 isoform, both implicated in synapse formation and plasticity (60, 61). Notably, polySia-NCAM was not significantly altered in the *Neu1* KO mice. The alterations in the MPS IIIC mouse models, therefore, most likely are not caused by the secondary NEU1 deficiency but related to neurodegeneration and impaired synaptic transmission observed in these mice (4, 14).

To conclude, the present study demonstrates that the secondary deficiency of NEU1 due to the HS-mediated dissociation of the LMC leading to oversialylation of glycoproteins in the brain tissues is a feature common of all neurological MPS diseases. Furthermore, our data show that NEU1 deficiency contributes to the CNS pathology in MPS diseases through defects in synaptic plasticity, yielding further insights into MPS pathophysiology and potentially suggesting new routes for therapeutic intervention.

## Methods

### Sex as a biological variable.

The study included samples from both male and female patient and iPSC-derived cultured neurons with sex-matched controls. Equal cohorts of male and female mice were studied separately for each experiment, and statistical methods were used to test whether the progression of the disease, levels of biomarkers, or response to therapy were different between male and female animals. Differences between sexes were not detected; therefore, the data for male and female mice and cells were pooled together. The findings are expected to be relevant to more than 1 sex.

### Methods.

The MPS IIIC knock-in model *Hgsnat^P304L^*, MPS IIIC KO model, *Hgsnat-Geo*, Tay-Sachs KO mouse model *hexa^–/–^*, and NEU1 KO mouse model *Neu1^–/–^* mouse models of MPS diseases (MPS I, II, IIIA, IIIB, and IVA) (12, 62–64) and other neurological LSDs (MLD, ML IV, and NPC1) (65–67) have been previously described (4, 14, 19, 68). All mice were housed under 12-hour light/dark cycles with ad libitum access to a normal rodent chow and water. Total acidic neuraminidase, NEU1, GLB1, total β-hexosaminidase, and GALNS were measured as previously described (16, 69, 70). SGSH activity was measured as described by Karpova et al. (71). Relative expression of *Neu1*, *Neu2*, *Neu3*, and *Neu4* in kidneys was determined by quantitative RT-PCR using previously described primers (19). Analysis of protein N-glycosylation by MALDI MS was conducted as previously described (72, 73). For the analysis of tissues by histochemistry, lectin histochemistry, and immunofluorescence, mice were anesthetized with sodium pentobarbital and fixed by intracardiac perfusion with 4% paraformaldehyde, and their tissues were processed essentially as described previously (74, 75). Analysis of GAGs in mouse brain tissues was conducted as previously described (7).

For complete methods, see Supplemental Methods and Supplemental Table 3.

### Statistics.

Statistical analyses were performed using Prism software (GraphPad Software). All data were analyzed for normal distribution using the Shapiro-Wilk normality test. Significance of the difference was determined using a 2-tailed *t* test (normal distribution) or Mann-Whitney test when comparing 2 groups. One-way ANOVA, nested 1-way ANOVA (normal distribution), or the Kruskal-Wallis test, followed by Dunn’s or Tukey’s multiple comparisons tests, were used when comparing more than 2 groups. Two-way ANOVA was used for 2-factor analysis. *P* ≤ 0.05 was considered significant.

### Study approval.

Ethical approval for research involving human tissues was given by the CHU Ste-Justine Research Ethics Board (Comité d’éthique de la recherche FWA00021692, approval 2020-2365). The NIH’s NeuroBioBank provided the cerebral tissues, frozen or fixed with paraformaldehyde, from patients with MPS and age-, ethnicity-, and sex-matched control participants (project 1071, MPS Synapse), along with clinical descriptions and the results of a neuropathological examination. Approval for the use of the animals in experimentation was granted by the Animal Care and Use Committee of the CHU Ste-Justine (approval 2023-4090).

### Data availability.

All data generated or analyzed during this study are included in this article, its supplemental information files, and the Supporting Data Values file.

## Author contributions

TMX, RHR, TM, PD, XP, TG, RJH, LS, AP, IR, AG, and HT conducted experiments and acquired data. TMX, RHR, TM, PD, LS, AP, DG, IR, HT, HH, CWC, TL, SK, AD, BG, and AVP analyzed data. BB, TMD, TL, JA, BA, CWC, and ST provided essential resources. AVP, DG, LS, HH, TMX, and RHR designed the experiments and wrote the first draft of the manuscript. BB, TMD, TL, JA, BA, CWC, BN, and AVP edited the manuscript. All authors read and approved the final version of the manuscript. The order of the 2 first authors’ names was determined by joint decision of these authors.

## Supplementary Material

Supplemental data

Unedited blot and gel images

Supporting data values

## Version 1 06/20/2025

In-Press Preview

## Version 2 08/15/2025

Electronic publication
