# Supplementary material for: Neuraminidase 1 secondary deficiency contributes to CNS pathology in neurological mucopolysaccharidoses via brain protein hypersialylation
Source: J Clin Invest. 2025 Jun 17;135(16):e177430. doi: 10.1172/JCI177430 (PMC12352893; doi:10.1172/JCI177430)
Supplement: Supplemental data [file jci-135-177430-s122.pdf]

Supplemental Data Materials and Methods

Sex as a biological variant

The study involved both male and female patient's samples and iPSC-derived cultured neurons with sex matching controls. Equal cohorts of male and female mice were studied separately for each experiment and statistical methods used to test whether the progression of the disease, levels of biomarkers or response to therapy were different between male and female animals. Since differences between sexes were not detected, the data for male and female mice and cells were pooled together. The findings are expected to be relevant to more than one sex.

Study approval.

Ethical approval for research involving human tissues was given by the CHU Ste-Justine Research Ethics Board (Comité d'éthique de la recherche FWA00021692, approval number 2020-2365). The National Institute of Health's NeuroBioBank provided the cerebral tissues, frozen or fixed with paraformaldehyde, from MPS patients as well as age, ethnicity and sex-matched controls (project 1071, MPS Synapse), along with clinical descriptions and the results of a neuropathological examination (see supplementary Table S1 for details). Approval for the use of the animals in experimentation was granted by the Animal Care and Use Committee of the CHU Ste-Justine (approval number 2023-4090).

Availability of data and materials

All data generated or analyzed during this study are included in this article and its supplementary information files.

Animals.

The MPS IIIC knock-in model *Hgsnat*^{P304L}, MPS IIIC knockout (KO) model, *Hgsnat-Geo*, Tay-Sachs KO mouse model *hexa*$^{-/-}$ and NEU1 KO mouse model *Neu1*$^{-/-}$ have been previously described [1-4]. The animals were housed in the Animal facilities of CHU Ste-Justine, following the guidelines of the Canadian Council on Animal Care (CCAC). The animals were kept in an enriched environment with a 12 h light/dark cycle, fixed temperature, humidity and continuous access to water and food. Frozen brains of previously described mouse models of MPS diseases (MPS I, II, IIIA, IIIB, IVA) [5-9], other neurological LSDs (MLD, ML IV, NPC1) [10-12] and their littermate controls were kindly provided by Drs Steven U. Walkley, Volkmar Gieselmann and Shunji Tomatsu. The mice were bred and maintained at the University of Manchester, Albert Einstein College of Medicine, the University of Bönn and the Thomas Jefferson University, Philadelphia.

Real time quantitative PCR.

Neu1, *Neu3* and *Neu4* mRNA was quantified using previously described primers [13] and a Stratagene Mx3000P QPCR system and mRNA isolated from homogenized half-brains of 2-month-old WT, *Hgsnat*^{P304L} and *Hgsnat*^{Geo} mice. Briefly, total mRNA was extracted using Trizol (Invitrogen), as described by the manufacturer, and reverse-transcribed using Quantitect reverse transcription kit (Qiagen) as per the manufacturer's protocol.

Enzyme activity assays.

Total acidic neuraminidase, NEU1, β-galactosidase, total β-hexosaminidase and GALNS were

measured as previously described [14-16], with minor modifications. Mice were anaesthetised with isoflurane and sacrificed using a CO_2 chamber. The brain, liver, spleen, kidneys and lungs were extracted and snap-frozen in liquid nitrogen, before being stored at -80 ºC. Approximately 50 mg of the tissue were homogenized in 250 µl of water using a sonic dismembrator (Artek Systems Corporation). The NEU1 activity reaction mixture (50 µL) containing 100 µg of total protein, NEU3/NEU4 inhibitor C-9-(4-biphenyl-triazolyl)-DANA (C9-4BPT-DANA; CG17700)[16] at a final concentration of 125 µM, and 4-methylumbelliferone-N-acetyl-neuraminic acid (Sigma-Aldrich) at a final concentration of 250 µM in 25 mM sodium acetate buffer, pH 4.6, was incubated at 37ºC for 60 minutes. The reaction was stopped with 950 µl of 0.4 M glycine buffer, pH 10.4. The concentration of the reaction product, 4-methylumbelliferone was measured using a ClarioStar plate reader (BMG Labtech). For all enzymatic assays, blank samples contained all components, except for the homogenate which was added after the termination of the reaction. The β-galactosidase activity reaction mixture containing 10 µl of homogenate, diluted 1:10 in ddH_2O, 12.5 µl of 0.4 M sodium acetate, 0.2 M sodium chloride buffer, pH 4.2, and 12.5 µl of the fluorogenic substrate 4-methylumbelliferyl β-D-galactoside (Sigma-Aldrich) was incubated at 37ºC for 15 min. The reaction was stopped with 965 µl of 0.4 M glycine buffer, pH 10.4 and the product measured as above. The β-hexosaminidase activity reaction mixture containing 2.5 µl of homogenate diluted 1:10, 15 µl of 0.1 M sodium acetate buffer, pH 4.2, and 12.5 µl of 4-methylumbelliferyl N-acetyl-β-D-glucosaminide (Sigma-Aldrich) was incubated for 15 minutes at 37°C. The reaction was stopped with 970 µl of 0.4 M glycine buffer, pH 10.4. The GALNS activity reaction mixture containing 25 µl of the homogenate, 12.5 µl of 1 M sodium acetate, 0.1 M acetic acid, 0.1 M sodium chloride, 5 mM lead acetate, 0.02% sodium azide buffer, pH 4.3, and 25 µl of 4-methylumbelliferyl β-D-galactopyranoside-6-sulphate (Toronto Research Chemicals) was

incubated for 3 h at 37°C. Then 12.5 μl of 1.8 M sodium phosphate, 0.02% sodium azide buffer was added, and the incubation continued at 37°C for another hour, when the reaction was stopped with 925 μl of 0.4 M glycine buffer, pH 10.4.

To measure SGSH enzyme activity in cultured iPSC and NPC cells, they were grown in T25 flasks to confluency, washed with 3-5 ml of a cold saline (0.9% sodium chloride) three times, placed on ice and collected with a rubber scraper. SGSH activity was measured as described by Karpova et al. [17], using the synthetic fluorogenic substrate, 4-methylumbelliferyl-2-Deoxy-2-sulfamino-α-D-glucopyranoside Na salt (4MU-α-GLcNs) in a 0.1 M Tris buffer, pH 6.5. Protein concentration was determined using Quick Start™ Bradford Protein Assay (Cat.# 5000201, Bio-Rad Hercules, CA).

Histochemistry and immunohistochemistry.

Sagittal 50 μm-thick sections of cryopreserved mouse brains were prepared as previously described [3]. To analyse bacterial LacZ/β-galactosidase reporter gene expression in the brains of *Neu1*$^{-/-}$ mice [3], the sections were washed with 0.1 M phosphate buffer, pH 7.3, supplemented with 2 mM $MgCl_2$ and stained with 0.1% (w/v) 5-bromo-4-chloro-3-indolyl-β-D-galactopyranoside (X-GAL) solution in 0.1 M phosphate buffer (pH 7.3) supplemented with 2 mM $MgCl_2$, 5 mM potassium ferrocyanide ($K_4Fe(CN)_6$-$3H_20$, Sigma-Aldrich cat. # P-9287) and 5 mM potassium ferricyanide ($K_3Fe(CN)_6$, Sigma-Aldrich cat. #P-8131) at 37°C overnight. The sections were further washed twice with 0.1 M phosphate buffer, pH 7.3 supplemented with 2 mM $MgCl_2$, mounted on the glass slides and images were acquired using a slide scanner (Axioscan 7, Zeiss).

For immunofluorescence, the brain sections were permeabilised and blocked using 0.3% (v/v)

Triton X-100, 5% (w/v) bovine serum albumin solution in phosphate-buffered saline (PBS), and stained with primary antibodies diluted in 1% bovine serum albumin, 0.3% Triton X-100 and PBS, overnight at 4ºC. This was followed by incubation with an appropriate Alexa Fluor-conjugated secondary antibodies (Life Technologies, see Table S3 for antibodies and dilutions). The slides were mounted using Prolong Gold Antifade Reagent with DAPI (Thermo Fisher Scientific) and images were acquired using a Leica TCS SPE inverted confocal microscope with a 63X oil objective and the same laser settings, exposure time and the number of Z-tacks across animals. Images for all groups of animals were acquired on the same day in the blind fashion.

Regions of interest were selected from the same cortical areas in the layers IV and V of the somatosensory cortex at the same distance from the dorsal hippocampus. For mice injected with LV, four regions of interest were selected, one from the same area in the dentate gyrus and three from the same areas of CA1. The percentage of stained area was quantified using the ImageJ software for immunohistochemical analysis. The images were z-stacked (z-project, max projection) and converted to B/W (8-bit) format using the same threshold. The percentages of stained areas were averaged within the same animal and compared between different animal groups using nested ANOVA. Densities of PSD-95, VGLUT1, and BDNF positive puncta were estimated by manually counting puncta along the axon at 30 μm increments starting from 10 μm away from the soma. The results were expressed as a mean numbers of puncta/10 μm.

Analysis of N-linked glycans and GAGs by mass spectrometry.

Proteins from homogenized mouse brains were washed with methanol/chloroform mixture to remove lipids and dried under a nitrogen stream as described [18,19]. Following the denaturation, reduction and alkylation of proteins, N-glycans were released by PNGase F treatment, permethylated and analysed by MALDI-TOF MS and MS/MS using a 4800 Proteomic Analyzer

instrument (AB Sciex) as described [19]. Analysis of GAGs in mouse brain tissues was conducted as previously described [20].

Analysis of urinary oligosaccharides by MALDI-TOF mass spectrometry.

Urine samples from three MPS IIIC and age-matched healthy controls were collected at CHU Ste-Justine under informed consent. Aliquots corresponding to 0.09 mg creatinine were processed as described [21]. The obtained glycans were permethylated and dissolved in MeOH/H_2O mixture containing 5-chloro-2-mercaptobenzothiazole matrix (10 mg/ml in 80:20 MeOH/H_2O, v/v) before the analysis on a UltrafleXtreme MALDI-TOF/TOF mass spectrometer (Bruker Daltonics, Germany).

Extraction of secreted HS oligosaccharides from MPS IIIC patients' urine.

Secreted HS oligosaccharides were isolated from MPS IIIC patients urine samples, obtained from the families with informed research consent. Thirty mL of urine was adjusted to a pH 5-6 with acetic acid. After 10 min centrifugation at 1050 g, the supernatant was collected and mixed with 600 µl of 5% cetylpyridinium chloride (CPC). The mixture was then incubated overnight at 4°C and centrifuged for 30 min at 1050 g and 4°C to collect the HS-CPC precipitate. The precipitate was washed twice with 12 mL of ethanol saturated with NaCl; each washing step was followed by 10 min centrifugation at 1050 g and 4°C. After removing the supernatant, 12 mL of 100% ethanol was added to the samples. Following centrifugation, the precipitate was allowed to dry before adding 3 mL of diethyl ether. The supernatant was removed, and the residue dried for another 30 min by a flow of N_2. Six mL of 0.6 M NaCl was then added to the precipitate, and the mixture was incubated for 3 h at 4°C. After incubation, the samples were cleared by centrifugation and mixed with 24 mL of 100% ethanol. After overnight incubation at 4°C, all steps were repeated and the

precipitate (4-5 mg of purified HS oligomers) resuspended in water, freeze-dried and stored at -20°C.

Analysis of HS treatment on NEU1 activity in cultured bone marrow-derived macrophages (BMDM).

Bone marrow cells (BMC) were collected from the tibia, femur, and iliac bones of WT and *Hgsnat*^{P304L} mice. After washing with 70% ethanol and ice-cold PBS containing 1% penicillin and streptomycin, the bones were flushed with ice-cold Dulbecco's Modified Eagle Medium (DMEM) at both sides to extract bone marrow cells. The cells were collected by centrifugation (10 min at 450 g and 4°C), and resuspended in a red blood cell lysis buffer (155 mM NH_4Cl, 12 mM $NaHCO_3$, and 0.1 M EDTA). After incubation for 30 sec, ice-cold complete DMEM was added to stop the lysis, BMC were collected by centrifugation as before and filtered using a 40 µm Nylon cell strainer. The cells were then cultured in 10 cm Petri dishes in DMEM supplemented with 10% fetal bovine serum (FBS) and containing granulocyte-stimulating factor, obtained from cultured L929 cells [22]. To study the effect of HS, BMDM were cultured for seven days in six-well plates at a density of 5 x 10^6 cells/well in the absence or presence of HS added in the concentration of 50, 100 and 300 of µg/mL after seeding and after three days in culture, when the medium was replaced. After seven days, the cells were washed with ice-cold PBS and harvested. The total neuraminidase and NEU1 activity were measured in cell homogenates as described above.

Analysis of brain protein extracts by size-exclusion chromatography.

Mouse brains (~1 g of tissue) were homogenized using a Polytron in 1 ml of 20 mM sodium acetate buffer containing 0.15 M NaCl and 1% Zwittergent 3-14 detergent (Millipore-Sigma 693017), pH

4.75, and cleared by centrifugation for 1 h at 100,000 *g*. A 0.5 ml aliquot of the supernatant was applied to a FPLC Superose 6 column (Pharmacia) and eluted with the same buffer containing 0.1% of Zwittergent at a flow rate of 0.4 ml/min. Thirty 0.5 ml fractions were collected and analysed for neuraminidase, NEU1 and β-galactosidase activities. The molecular masses of the eluted proteins were determined using the calibration curve obtained with the following MW standards (Pharmacia): blue dextran (~2,000 kDa), thyroglobulin (669 kDa), ferritin (440 kDa), catalase (232 kDa), aldolase (158 kDa), BSA (69 kDa), chymotrypsinogen (25 kDa), and ribonuclease A (13.7 kDa).

In vitro analysis of HS effects on NEU1 enzymatic activity and solubility.

Human NEU1, GLB1 and CTSA were expressed as secreted proteins in Sf9 insect cells, infected with recombinant baculovirus, and purified as previously described [23,24], except that a TEV protease cleavage site was included between the hexahistidine tag and the catalytic domains of NEU1 and CTSA proteins. NEU1 enzymatic activity assays were carried out in 50 mM sodium acetate buffer, pH 4.5, with 100 mM NaCl and 1 mM of the fluorogenic substrate 4-methylumbelliferyl N-acetyl-α-D-neuraminic acid (4MU-NANA, BioSynth EM05195). 20 μM (approximately 1 mg/mL) of TEV-cleaved CTSA was pre-incubated with varying concentrations of HS (sodium salt, from bovine kidney, Sigma H7640) for 30 min at 22 ˚C, followed by addition of 20 nM of TEV-cleaved NEU1 and incubation for 30 min. After addition of the substrate and incubation for 30 min at 37 ˚C, the reaction was stopped with 200 mM glycine, pH 10, and the fluorescence of the product 4-methylumbelliferone measured as described above.

To analyze HS effect on the protein solubility, purified recombinant human CTSA, GLB1 or NEU1 were incubated at equimolar amounts (1, 1.41 or 0.79 mg/mL respectively) in the presence

of HS (Selleck Chemicals S5992, final concentration 1 mg/mL) in 25 mM sodium acetate buffer, pH 4.5, with 100 mM NaCl for 30 min at 22 ˚C. Samples were, then, centrifuged at 21000 g for 5 min and pellets solubilized in 8 M urea. Supernatants and pellets were analyzed by SDS-PAGE. CTSA is partially processed into a 30 kDa large and 18 kDa small protein chains by trace amount of endogenous Sf9 proteases during the purification.

Molecular docking.

Coordinates for NEU1 were from the PDB (8DU5)[23]. Initial coordinates for HS tetramers were generated from previous reports [25]. Autodock Vina (version 1.2.5) was used to generate and score docked poses [26], with input files generated in Webina [27]. Poses of heparan sulfate tetramers were then inspected and figures were generated in ChimeraX [28].

Analyses of polySia-NCAM.

Frozen brain samples comprising the right anterior cerebral cortex of 4-month-old WT, *Neu1*$^{-/-}$, *Hgsnat*^{P304L}, and *Hgsnat-Geo* mice with average weights of 15.7 mg were homogenized in 30 µl ice cold lysis buffer (100 mM Tris-HCl, pH 7.4, 2 mM EDTA, 1% NP40, 1 mM phenylmethylsulfonyl fluoride, 14 µg/ml aprotinin) per mg sample weight. Lysates were clarified by centrifugation (15 min, 15.700 rcf, 4°C) and total protein concentrations were determined using the Bio-Rad protein assay. Levels of polysialylated neural cell adhesion molecule (polySia-NCAM) were determined by a sandwich ELISA [29] adapted to mouse samples. Briefly, 96 well half area microplates (Cat.# 675101, Greiner Bio-one, Frickenhausen, Germany) were coated with polysialic acid (polySia)-specific monoclonal antibody 735 [30] (mouse IgG_{2a}; 5 µg/ml in PBS, 25µl per well) for 2 h at room temperature (RT), washed with 0.1 M phosphate buffer, pH 7.4

containing 0.1% Tween20, and blocked overnight at 4°C with 1% bovine serum albumin (BSA, Merck, Cat# 7906) in PBS (200 µl per well). Ten µl of cortex lysate, diluted to a concentration of 50 µg of protein per ml, was added to each well, and the plates were incubated overnight at 4°C. After washing, wells were incubated with the NCAM specific monoclonal antibody OB11 (Merck, Cat.# C9672, mouse IgG_1; 1:1000), followed by horseradish peroxidase (HRP)-conjugated anti-mouse IgG_1 (Jackson Cat.# 115-035-205; 40 ng/ml), both for 1h at RT with 10 µl per well in blocking buffer). HRP substrate 3,3',5,5'-Tetramethylbenzidin (TMB, Merck, Cat.# T2885; 0.1 mg/ml in citrate buffer, pH 4.9, with 0.001% H_2O_2) was added, the reaction was stopped after 25 min with 1 M H_2SO_4 (10 µl per well), and absorbances were measured at 450 nm against 550 nm as a reference wavelength. Each sample was analyzed by four independent experiments and values were normalized to the mean value of the WT samples. Sample treated with endosialidase (6 µg/ml, 40 min at 37°C), degrading polySia with high specificity [31], were used as negative ELISA controls. Endosialidase treatment reduced absorption levels to <1% of the values for untreated samples. Western blot analysis of cortex lysates was performed with polySia-specific mAb 735 and NCAM-specific mAb H28 as described previously [32]. Detection and densitometric quantification were performed with the Odyssey Infrared Imaging System and Image studio 4.0.21 software (LI-COR Biosciences, Bad Homburg, Germany).

Generation and maintenance of induced pluripotent stem cells (iPSCs).

The induced pluripotent stem cells (iPSCs) were generated from the fibroblasts of an MPS IIIA patient, obtained from the Coriell Institute for Medical Research, NJ, USA (line GM01881), and a healthy sex and age-matched control from the biobank of CHUSJ. The generation and use of human iPSCs was approved by the Research Ethics Committee of CHUSJ (approval number

2022-3817). iPSCs were tested for mycoplasma and reprogrammed at CHUSJ Cell reprogramming and genome editing core using the CytoTune™-iPS 2.0 Sendai Kit (Life Technologies) [33]. Once generated, the iPSCs lines were maintained in a 5% CO2, 5% O_2, 37 °C incubator using Matrigel-coated dishes and mTeSR™ Plus (StemCell) media as described [34]. The expression of pluripotency markers Oct4, SSEA4, TRA-160, and SOX2 in all iPSCs lines was confirmed by immunofluorescence.

Generation of cultured cortical neuronal progenitor cells and cultured cortical iPSC-derived neurons.

After two passages, iPSCs were differentiated into a monolayer of forebrain committed neural progenitor cells (NPCs) by dual SMAD inhibition, with FGF-8 used instead of FGFb-2 [35]. NPC were cultured using a neuronal induction media (DMEM/F12) for 3 weeks and analyzed by immunofluorescence to confirm the expression of the endoderm markers, Nestin and PAX6, as well as the neuronal markers, NeuN, axonal β-tubulin III (clone TUJ1) and Syn1. After three weeks in culture, NPC were further differentiated into cortical-specific neurons as described [36] but MPS IIIA patient cell lines were transduced with the LV-CTSA-IRES-NEU1-GFP or control LV-GFP virus at a multiplicity of infection of 10 (MOI 10) for 24 h before the switch to the differentiation media. Neurons were then cultured to day 28 to achieve a complete differentiation and maturation. All cells expressed fidelity markers Nestin/Pax6/Tuj1/Syn1) as well as the cortical specific marker T-box brain 1 (TBR1). Fluorescence activated cell sorting (FACS) analysis confirmed that >80% of cells were TBR1-positive and ~72% of cells were NeuN/ TBR1-positive, demonstrating a high degree of conversion. The primary enzymatic deficiency of SGSH in MPS IIIA cells was confirmed at both iPSC and NPC stages. Size and abundance of

LAMP2-positive perinuclear puncta, a marker for lysosomal storage/increased lysosomal biogenesis phenotype [37], were measured by immunocytochemistry at the NPC stage.

Immunoblot

Mouse brain samples (~200 mg) were homogenized 1:5 (w/v) in a buffer containing 50 mM sodium acetate (pH 5.0), 100 mM NaCl, 1 mM $CaCl_2$, 1 mM $MgCl_2$, 1 mM $MnCl_2$, and 1% Zwittergent detergent. After 2 h of rocking at 4°C, the lysates were cleared by centrifugation at 13000xg for 20 min at 4°C. To enrich glycoproteins, concanavalin A (Con-A) Sepharose beads (Pharmacia Biotech, catalog #17-0440-01) were washed in the homogenization buffer. One milliliter of supernatant was incubated with Con-A beads at 4°C overnight under gentle rotation. After centrifugation (2 min at 5,000×g), the beads were washed three times with the same buffer. Then 50 µL of 1X Laemmli buffer was added to the pellets, and the samples were vortexed, boiled for 5 min, and centrifuged for 5 min at 10,000xg. Twenty microliters of the supernatant was loaded onto a 12% SDS-PAGE gel; proteins were separated by electrophoresis and transferred onto a nitrocellulose membrane. Membranes were blocked with blocking solution (5% BSA in TBST) for 1 h at room temperature. Membranes were incubated overnight at 4°C with a primary anti-NEU1 rabbit monoclonal antibody (ABclonal #A25845, 1:1000 in 5% BSA/TBST), followed by incubation with an HRP-conjugated anti-rabbit IgG antibody at a 1:10000 dilution. Signal detection was performed using ECL reagent and imaged with the ChemiDoc system (Bio-Rad). Densitometric analysis was carried out using Image Lab. Total protein normalization was performed using Ponceau Red stained membranes. Immunoblot of CTSA was performed in total brain extracts using affinity-purified polyclonal goat antibodies against human CTSA protein (R&D Systems, #AF1049)

Stereotaxic injections of mice with lentiviral vectors.

The previously described [38] CTSA-IRES-NEU1-GFP biscistronic HIV-1-based recombinant vesicular stomatitis virus glycoprotein-pseudotyped (VSVg) LV and control GFP-LV were generously provided by Dr. Jeffrey A Medin (Medical College of Wisconsin, Milwaukee, USA). LV titers were 1.4 × 10^8 TU/ml for CTSA-IRES-NEU1-GFP LV and 8.3 × 10^8 TU/ml for control NEU1-GFP LV. Postnatal days 18 and 19 (P18, P19) mice were anesthetized with 5% isoflurane and oxygen in an induction chamber. During the surgery, mice remained on a heating pad to prevent hypothermia and the mouse's head was secured in an induction cone with a flow of 2 L of oxygen containing 2% isoflurane per min. Mice were further immobilized by fixing their heads with ear bars and injected subcutaneously with a non-steroidal anti-inflammatory drug Carprofen/Rimadyl (Zoetis, DIN 02255693, 0.1 mL of Carprofen mixed with 9.9 mL of 0.9% Sodium Chloride, 0.15 mL per mouse) and a lubricant OptixCare was applied to protect the eyes. After removal of the fur on top of the head and disinfecting the surgical area with 10% povidone-iodine solution (Laboratoire Atlas) the skin was cut open. One hole was made on each side of the brain using a microdrill (Foredom, K.1070 Micromotor kit). The coordinates were 1.5 mm on each hemisphere's M/L axis and 2.2 mm on the A/P axis. The holes were less than 1 mm in diameter and deep enough to pierce the skull without damaging the dura mater. To remove bone fragments around the holes, a saline solution was used to flush the surrounding areas.

The glass capillary needles for injections were prepared using glass capillaries (3.5" Drummond #3-000-203-G/X) and a micropipette puller (Sutter Instrument Company). After backfilling the glass capillary needles with mineral oil (Sigma BioReagent, #MKCM5718), the needles were fixed onto the Nanoliter Injector (Drummond Scientific Company, Nanoject III #3-000-207) and the Nanoliter micropipette was moved to the injection site (1.5 mm M/L, 2.2 mm A/P). For each

hemisphere, the micropipette was first lowered to a depth of 2.2 mm to target the hippocampus, and then, to 1.2 mm to target layers IV and V of the cortex. The LV was injected at a speed of 2 nL/second using the Nanoliter injector, and each injection lasted about seven minutes. After injecting 900 nL of the virus into the hippocampus, we waited six minutes for the viral solution to set down before moving the needle to the cortex. After all the injections were completed, the wound was sutured with ethilon nylon suture (Ethicon, PMP346), and an antibiotic cream (Polysporin, Jonhson & Jonhson inc, DIN 02237227), was applied to the surgical zone. Finally, another dose of diluted Carprofen (0.15 mL) was injected subcutaneously into the mouse, which was then removed from the stereotaxic frame and returned to its cage. All the injected mice were put on a wet diet, and their condition was observed daily following the surgery.

Behavioural analysis.

Novel object recognition (NOR) test was used to measure short-term memory. One day before the experiment, mice were habituated to a white plexiglass box (45 cm length x 45 cm width x 40 cm height) for 10 min and, then, returned to their home cages. The next day, mice were placed individually in the testing chamber facing the wall at the opposite side of two identical blue plastic cylinders measuring 6.5 x 7.5 cm and placed at a 15 cm distance from the walls. The animals were allowed to explore the objects for 10 min before returning to their home cages. One hour later, the mouse was placed back into the testing chamber, which contained one of the original and a new object, a red plastic cube measuring 6.5 x 6.5 x 6.5 cm. Each trial with the novel object lasted for 10 min. Between each session, the testing chambers and the objects were washed with 70% ethanol to eliminate any olfactory cue bias. Each session was video-recorded and analyzed manually by an operator blinded to the animal genotype and treatment. The exploration time was counted when

the head of the mouse was within a 3 cm radius of the object, and when the animal was looking at the object, sniffing the object, or touching the object with its snout. Exploration time was not counted when the mouse was within the exploration zone but not actively exploring the objects, or if the animal was grooming itself or sitting on top of the toys. The discrimination index (DI) was calculated as the difference in time spent exploring the new and original objects divided by the total exploration time.

Open field (OF) test was used to measure anxiety and activity as described [39]. Mice were habituated to the testing room for 30 min before the experiment, placed in the center of an open-field arena (45 cm length x 45 cm width x 40 cm height) under dimed light conditions (~30 lux) and allowed to explore it for 20 min. Each session was recorded and analyzed by the Smart 3.0 software. The following parameters were measured: the number of entries in the center, the total distance traveled, the percent of time spent in the center zone, and the distance traveled in the center zone. The arena was cleaned with 70% ethanol between the trials. OFT was always performed one hour into the mouse light cycle by the same investigator (TM.X.).

Statistical analysis.

Statistical analyses were performed using Prism GraphPad software (GraphPad SoftwareSan Diego, CA). All data were analyzed for normal distribution using the Shapiro-Wilk normality test. Significance of the difference was determined using t-test (normal distribution) or Mann-Whitney test when comparing two groups. One-way ANOVA, nested ANOVA (normal distribution) or Kruskal-Wallis test followed by Dunn or Tukey multiple comparisons tests were used when comparing more than two groups. Two-way ANOVA was used for two-factor analysis. A P-value of 0.05 or less was considered significant.

1. Pan, X.; Taherzadeh, M.; Bose, P.; Heon-Roberts, R.; Nguyen, A.L.A.; Xu, T.; Para, C.; Yamanaka, Y.; Priestman, D.A.; Platt, F.M., et al. Glucosamine amends CNS pathology in mucopolysaccharidosis IIIC mouse expressing misfolded HGSNAT. *J Exp Med* **2022**, *219*, doi:10.1084/jem.20211860.
2. Martins, C.; Hůlková, H.; Dridi, L.; Dormoy-Raclet, V.; Grigoryeva, L.; Choi, Y.; Langford-Smith, A.; Wilkinson, F.L.; Ohmi, K.; DiCristo, G., et al. Neuroinflammation, mitochondrial defects and neurodegeneration in mucopolysaccharidosis III type C mouse model. *Brain* **2015**, *138*, 336-355, doi:10.1093/brain/awu355.
3. Pan, X.; De Aragao, C.B.P.; Velasco-Martin, J.P.; Priestman, D.A.; Wu, H.Y.; Takahashi, K.; Yamaguchi, K.; Sturiale, L.; Garozzo, D.; Platt, F.M., et al. Neuraminidases 3 and 4 regulate neuronal function by catabolizing brain gangliosides. *FASEB journal : official publication of the Federation of American Societies for Experimental Biology* **2017**, 10.1096/fj.201601299R, doi:10.1096/fj.201601299R.
4. Seyrantepe, V.; Lema, P.; Caqueret, A.; Dridi, L.; Bel Hadj, S.; Carpentier, S.; Boucher, F.; Levade, T.; Carmant, L.; Gravel, R.A., et al. Mice Doubly-Deficient in Lysosomal Hexosaminidase A and Neuraminidase 4 Show Epileptic Crises and Rapid Neuronal Loss. *PLOS Genetics* **2010**, *6*, e1001118, doi:10.1371/journal.pgen.1001118.
5. Clarke, L.A.; Russell, C.S.; Pownall, S.; Warrington, C.L.; Borowski, A.; Dimmick, J.E.; Toone, J.; Jirik, F.R. Murine mucopolysaccharidosis type I: targeted disruption of the murine alpha-L-iduronidase gene. *Hum Mol Genet* **1997**, *6*, 503-511, doi:10.1093/hmg/6.4.503.
6. Muenzer, J.; Lamsa, J.C.; Garcia, A.; Dacosta, J.; Garcia, J.; Treco, D.A. Enzyme replacement therapy in mucopolysaccharidosis type II (Hunter syndrome): a preliminary report. *Acta Paediatr Suppl* **2002**, *91*, 98-99, doi:10.1111/j.1651-2227.2002.tb03115.x.
7. Bhaumik, M.; Muller, V.J.; Rozaklis, T.; Johnson, L.; Dobrenis, K.; Bhattacharyya, R.; Wurzelmann, S.; Finamore, P.; Hopwood, J.J.; Walkley, S.U., et al. A mouse model for mucopolysaccharidosis type III A (Sanfilippo syndrome). *Glycobiology* **1999**, *9*, 1389-1396.
8. Li, H.H.; Yu, W.H.; Rozengurt, N.; Zhao, H.Z.; Lyons, K.M.; Anagnostaras, S.; Fanselow, M.S.; Suzuki, K.; Vanier, M.T.; Neufeld, E.F. Mouse model of Sanfilippo syndrome type B produced by targeted disruption of the gene encoding alpha-N-acetylglucosaminidase. *Proc Natl Acad Sci U S A* **1999**, *96*, 14505-14510.
9. Tomatsu, S.; Orii, K.O.; Vogler, C.; Nakayama, J.; Levy, B.; Grubb, J.H.; Gutierrez, M.A.; Shim, S.; Yamaguchi, S.; Nishioka, T., et al. Mouse model of N-acetylgalactosamine-6-sulfate sulfatase deficiency (Galns-/-) produced by targeted disruption of the gene defective in Morquio A disease. *Hum Mol Genet* **2003**, *12*, 3349-3358, doi:10.1093/hmg/ddg366.
10. Hess, B.; Saftig, P.; Hartmann, D.; Coenen, R.; Lullmann-Rauch, R.; Goebel, H.H.; Evers, M.; von Figura, K.; D'Hooge, R.; Nagels, G., et al. Phenotype of arylsulfatase A-deficient mice: relationship to human metachromatic leukodystrophy. *Proc Natl Acad Sci U S A* **1996**, *93*, 14821-14826, doi:10.1073/pnas.93.25.14821.
11. Matthes, F.; Andersson, C.; Stein, A.; Eistrup, C.; Fogh, J.; Gieselmann, V.; Wenger, D.A.; Matzner, U. Enzyme replacement therapy of a novel humanized mouse model of

globoid cell leukodystrophy. *Exp Neurol* **2015**, *271*, 36-45, doi:10.1016/j.expneurol.2015.04.020.

12. Maue, R.A.; Burgess, R.W.; Wang, B.; Wooley, C.M.; Seburn, K.L.; Vanier, M.T.; Rogers, M.A.; Chang, C.C.; Chang, T.Y.; Harris, B.T., et al. A novel mouse model of Niemann-Pick type C disease carrying a D1005G-Npc1 mutation comparable to commonly observed human mutations. *Hum Mol Genet* **2012**, *21*, 730-750, doi:10.1093/hmg/ddr505.
13. Pan, X.; De Aragao, C.B.P.; Velasco-Martin, J.P.; Priestman, D.A.; Wu, H.Y.; Takahashi, K.; Yamaguchi, K.; Sturiale, L.; Garozzo, D.; Platt, F.M., et al. Neuraminidases 3 and 4 regulate neuronal function by catabolizing brain gangliosides. *FASEB J* **2017**, *31*, 3467-3483, doi:10.1096/fj.201601299R.
14. Seyrantepe, V.; Canuel, M.; Carpentier, S.; Landry, K.; Durand, S.; Liang, F.; Zeng, J.; Caqueret, A.; Gravel, R.A.; Marchesini, S., et al. Mice deficient in Neu4 sialidase exhibit abnormal ganglioside catabolism and lysosomal storage. *Hum Mol Genet* **2008**, *17*, 1556-1568, doi:10.1093/hmg/ddn043.
15. Potier, M.; Mameli, L.; Bélisle, M.; Dallaire, L.; Melançon, S.B. Fluorometric assay of neuraminidase with a sodium (4-methylumbelliferyl-α-d-N-acetylneuraminate) substrate. *Analytical Biochemistry* **1979**, *94*, 287-296, doi:https://doi.org/10.1016/0003-2697(79)90362-2.
16. Guo, T.; Dätwyler, P.; Demina, E.; Richards, M.R.; Ge, P.; Zou, C.; Zheng, R.; Fougerat, A.; Pshezhetsky, A.V.; Ernst, B., et al. Selective Inhibitors of Human Neuraminidase 3. *Journal of Medicinal Chemistry* **2018**, *61*, 1990-2008, doi:10.1021/acs.jmedchem.7b01574.
17. Karpova, E.A.; Voznyi Ya, V.; Keulemans, J.L.; Hoogeveen, A.T.; Winchester, B.; Tsvetkova, I.V.; van Diggelen, O.P. A fluorimetric enzyme assay for the diagnosis of Sanfilippo disease type A (MPS IIIA). *J Inherit Metab Dis* **1996**, *19*, 278-285, doi:10.1007/bf01799255.
18. Sturiale, L.; Barone, R.; Garozzo, D. The impact of mass spectrometry in the diagnosis of congenital disorders of glycosylation. *J Inherit Metab Dis* **2011**, *34*, 891-899, doi:10.1007/s10545-011-9306-8.
19. Palmigiano, A.; Barone, R.; Sturiale, L.; Sanfilippo, C.; Bua, R.O.; Romeo, D.A.; Messina, A.; Capuana, M.L.; Maci, T.; Le Pira, F., et al. CSF N-glycoproteomics for early diagnosis in Alzheimer's disease. *J Proteomics* **2016**, *131*, 29-37, doi:10.1016/j.jprot.2015.10.006.
20. Viana, G.M.; Priestman, D.A.; Platt, F.M.; Khan, S.; Tomatsu, S.; Pshezhetsky, A.V. Brain Pathology in Mucopolysaccharidoses (MPS) Patients with Neurological Forms. *J Clin Med* **2020**, *9*, doi:10.3390/jcm9020396.
21. Xia, B.; Asif, G.; Arthur, L.; Pervaiz, M.A.; Li, X.; Liu, R.; Cummings, R.D.; He, M. Oligosaccharide analysis in urine by maldi-tof mass spectrometry for the diagnosis of lysosomal storage diseases. *Clin Chem* **2013**, *59*, 1357-1368, doi:10.1373/clinchem.2012.201053.
22. Heap, R.E.; Marin-Rubio, J.L.; Peltier, J.; Heunis, T.; Dannoura, A.; Moore, A.; Trost, M. Proteomics characterisation of the L929 cell supernatant and its role in BMDM differentiation. *Life Sci Alliance* **2021**, *4*, doi:10.26508/lsa.202000957.
23. Gorelik, A.; Illes, K.; Mazhab-Jafari, M.T.; Nagar, B. Structure of the immunoregulatory sialidase NEU1. *Sci Adv* **2023**, *9*, eadf8169, doi:10.1126/sciadv.adf8169.

24. Gorelik, A.; Illes, K.; Hasan, S.M.N.; Nagar, B.; Mazhab-Jafari, M.T. Structure of the murine lysosomal multienzyme complex core. *Sci Adv* **2021**, *7*, doi:10.1126/sciadv.abf4155.
25. Wander, R.; Kaminski, A.M.; Xu, Y.; Pagadala, V.; Krahn, J.M.; Pham, T.Q.; Liu, J.; Pedersen, L.C. Deciphering the substrate recognition mechanisms of the heparan sulfate 3-O-sulfotransferase-3. *RSC Chem Biol* **2021**, *2*, 1239-1248, doi:10.1039/d1cb00079a.
26. Eberhardt, J.; Santos-Martins, D.; Tillack, A.F.; Forli, S. AutoDock Vina 1.2.0: New Docking Methods, Expanded Force Field, and Python Bindings. *J Chem Inf Model* **2021**, *61*, 3891-3898, doi:10.1021/acs.jcim.1c00203.
27. Kochnev, Y.; Hellemann, E.; Cassidy, K.C.; Durrant, J.D. Webina: an open-source library and web app that runs AutoDock Vina entirely in the web browser. *Bioinformatics* **2020**, *36*, 4513-4515, doi:10.1093/bioinformatics/btaa579.
28. Pettersen, E.F.; Goddard, T.D.; Huang, C.C.; Meng, E.C.; Couch, G.S.; Croll, T.I.; Morris, J.H.; Ferrin, T.E. UCSF ChimeraX: Structure visualization for researchers, educators, and developers. *Protein Sci* **2021**, *30*, 70-82, doi:10.1002/pro.3943.
29. Piras, F.; Schiff, M.; Chiapponi, C.; Bossu, P.; Muhlenhoff, M.; Caltagirone, C.; Gerardy-Schahn, R.; Hildebrandt, H.; Spalletta, G. Brain structure, cognition and negative symptoms in schizophrenia are associated with serum levels of polysialic acid-modified NCAM. *Transl Psychiatry* **2015**, *5*, e658, doi:10.1038/tp.2015.156.
30. Frosch, M.; Gorgen, I.; Boulnois, G.J.; Timmis, K.N.; Bitter-Suermann, D. NZB mouse system for production of monoclonal antibodies to weak bacterial antigens: isolation of an IgG antibody to the polysaccharide capsules of Escherichia coli K1 and group B meningococci. *Proc Natl Acad Sci U S A* **1985**, *82*, 1194-1198, doi:10.1073/pnas.82.4.1194.
31. Schulz, E.C.; Dickmanns, A.; Urlaub, H.; Schmitt, A.; Muhlenhoff, M.; Stummeyer, K.; Schwarzer, D.; Gerardy-Schahn, R.; Ficner, R. Crystal structure of an intramolecular chaperone mediating triple-beta-helix folding. *Nat Struct Mol Biol* **2010**, *17*, 210-215, doi:10.1038/nsmb.1746.
32. Tantra, M.; Krocher, T.; Papiol, S.; Winkler, D.; Rockle, I.; Jatho, J.; Burkhardt, H.; Ronnenberg, A.; Gerardy-Schahn, R.; Ehrenreich, H., et al. St8sia2 deficiency plus juvenile cannabis exposure in mice synergistically affect higher cognition in adulthood. *Behav Brain Res* **2014**, *275*, 166-175, doi:10.1016/j.bbr.2014.08.062.
33. Kawamura, T.; Suzuki, J.; Wang, Y.V.; Menendez, S.; Morera, L.B.; Raya, A.; Wahl, G.M.; Izpisua Belmonte, J.C. Linking the p53 tumour suppressor pathway to somatic cell reprogramming. *Nature* **2009**, *460*, 1140-1144, doi:10.1038/nature08311.
34. Chen, X.; Rocha, C.; Rao, T.; Durcan, T. NeuroEDDU protocols_iPSC culture_interactive protocol. **2019**, 10.5281/ZENODO.3733913, doi:10.5281/ZENODO.3733913.
35. Chen, X.; Rocha, C.; Loignon, M.; Peng, H.; Rao, T.; Durcan, T.M. Induction of Dopaminergic or Cortical neuronal progenitors from iPSCs. **2019**, 10.5281/ZENODO.3738358, doi:10.5281/ZENODO.3738358.
36. Chen, X.; Lauinger, N.; Rocha, C.; Rao, T.; Durcan, T.M. Generation of dopaminergic or cortical neurons from neuronal progenitors (interactive protocol). **2019**, 10.5281/ZENODO.3733914, doi:10.5281/ZENODO.3733914.
37. Mooree, T.; Bose, P.; Wood, J.; Durcan, T.; Pshezhetsky, A. iPSC derived neurons of mucopolysaccharidosis III patients show pronounced synaptic defects. *Molecular*

Genetics and Metabolism **2022**, *135*, S85, doi:https://doi.org/10.1016/j.ymgme.2021.11.219.
38. Fougerat, A.; Pan, X.; Smutova, V.; Heveker, N.; Cairo, C.W.; Issad, T.; Larrivee, B.; Medin, J.A.; Pshezhetsky, A.V. Neuraminidase 1 activates insulin receptor and reverses insulin resistance in obese mice. *Mol Metab* **2018**, *12*, 76-88, doi:10.1016/j.molmet.2018.03.017.
39. Martins, C.; Hulkova, H.; Dridi, L.; Dormoy-Raclet, V.; Grigoryeva, L.; Choi, Y.; Langford-Smith, A.; Wilkinson, F.L.; Ohmi, K.; DiCristo, G., et al. Neuroinflammation, mitochondrial defects and neurodegeneration in mucopolysaccharidosis III type C mouse model. *Brain* **2015**, *138*, 336-355, doi:10.1093/brain/awu355.

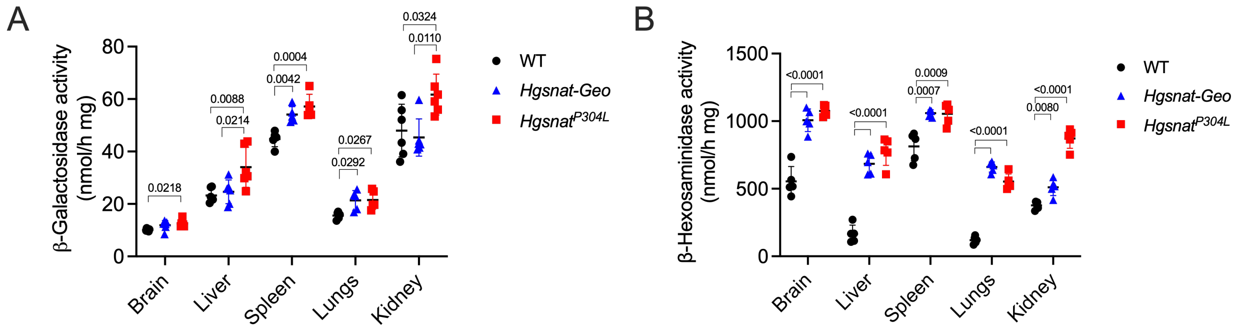

Figure S1. Increased lysosomal biogenesis in tissues of MPS IIIC mouse models.

Specific activities of β-galactosidase **(A)** and total β-hexosaminidase **(B)** were measured using the fluorogenic substrates, 4-methylumbelliferone-β-D-galactoside and 4-methylumbelliferyl-β-D-glucosaminide, respectively. The activities of both enzymes in tissues *Hgsnat-Geo* and *Hgsnat*^{P304L} mice are increased or show a trend for an increase compared to WT counterparts. Individual data and means ±SD (n=5) are shown. P values were calculated by one-way ANOVA with a Tukey post hoc test.

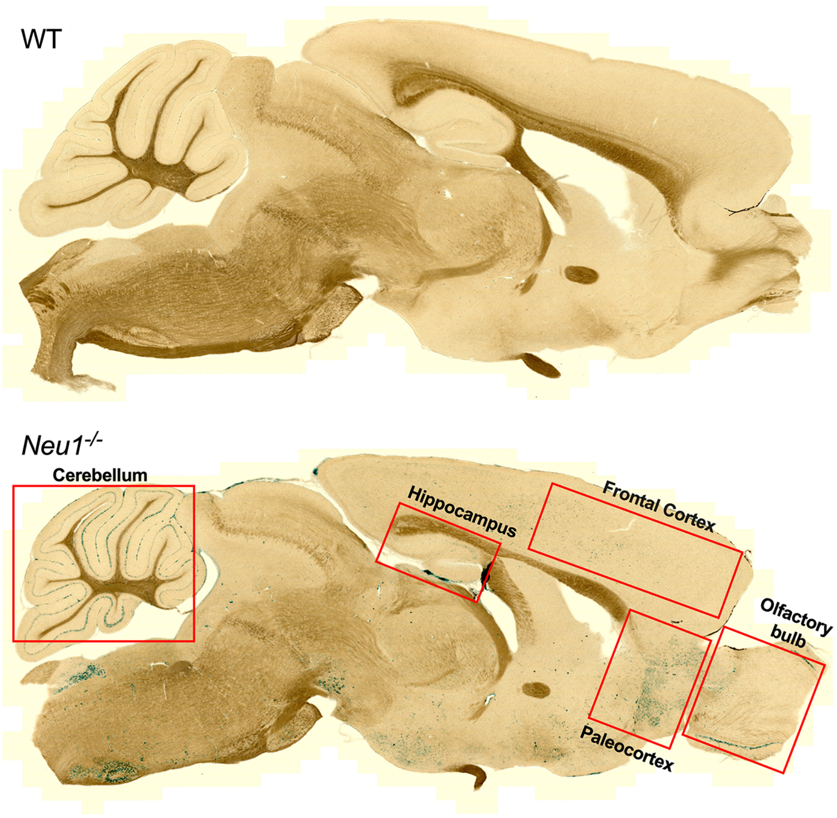

Figure S2. X-GAL staining of mouse brain sections.

Sagittal brain sections from 2-month-old WT and *Neu1*$^{-/-}$ mice were stained with the 5-bromo-4-chloro-3-indolyl-ß-D-galactopyranoside (X-GAL) substrate (blue) to visualise areas with high *Neu1* expression. The red boxes indicate regions that were dissected in WT, *Hgsnat*^{304L} and *Hgsnat-Geo* mice for NEU1 enzyme activity assays. Images were taken at 10x magnification using an AxioScan instrument.

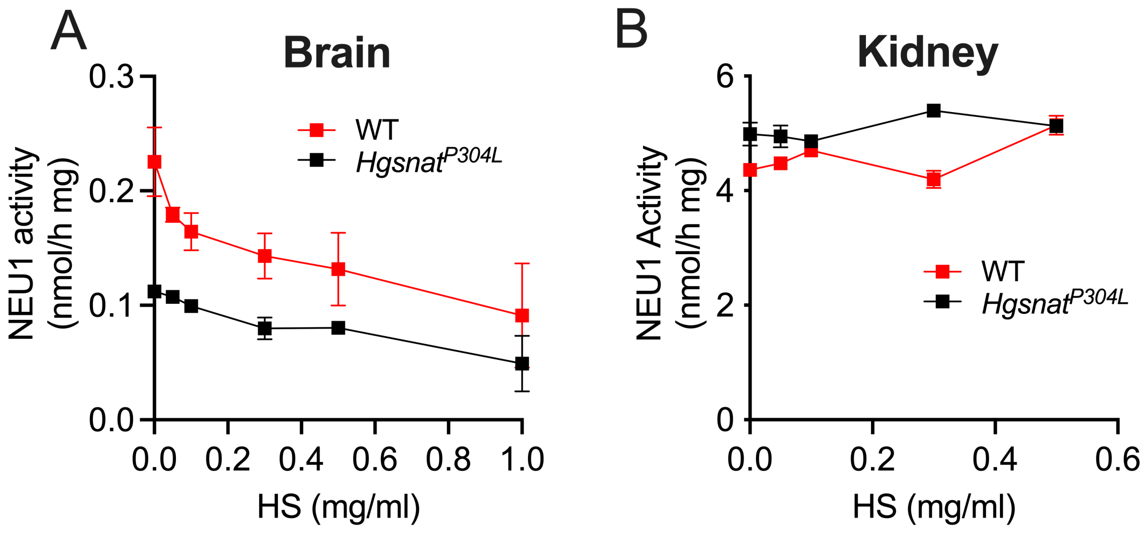

Figure S3. Treatment of murine brain homogenates with exogenous HS oligomers causes inhibition of NEU1 enzymatic activity.

Brain homogenates of WT and *Hgsnat*^{P304L} mice **(A)** and a kidney homogenate of a WT mice **(B)** were treated with HS oligomers before measuring NEU1 activity at pH 4.75. Graphs show means and SD of two independent experiments.

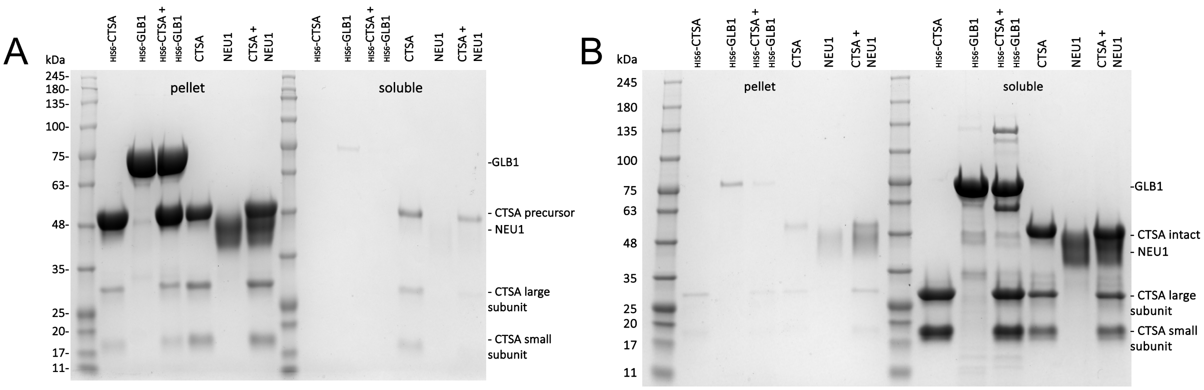

Figure S4. HS causes precipitation of NEU1, CTSA and GLB1 *in vitro*.

Purified recombinant NEU1, CTSA and GLB1 were incubated in various combinations **(A)** in presence of 1 mg/mL of HS or **(B)** in the absence of HS at pH 4.5 followed by centrifugation. Equal volumes of soluble and precipitated protein fractions were analyzed by SDS-PAGE.

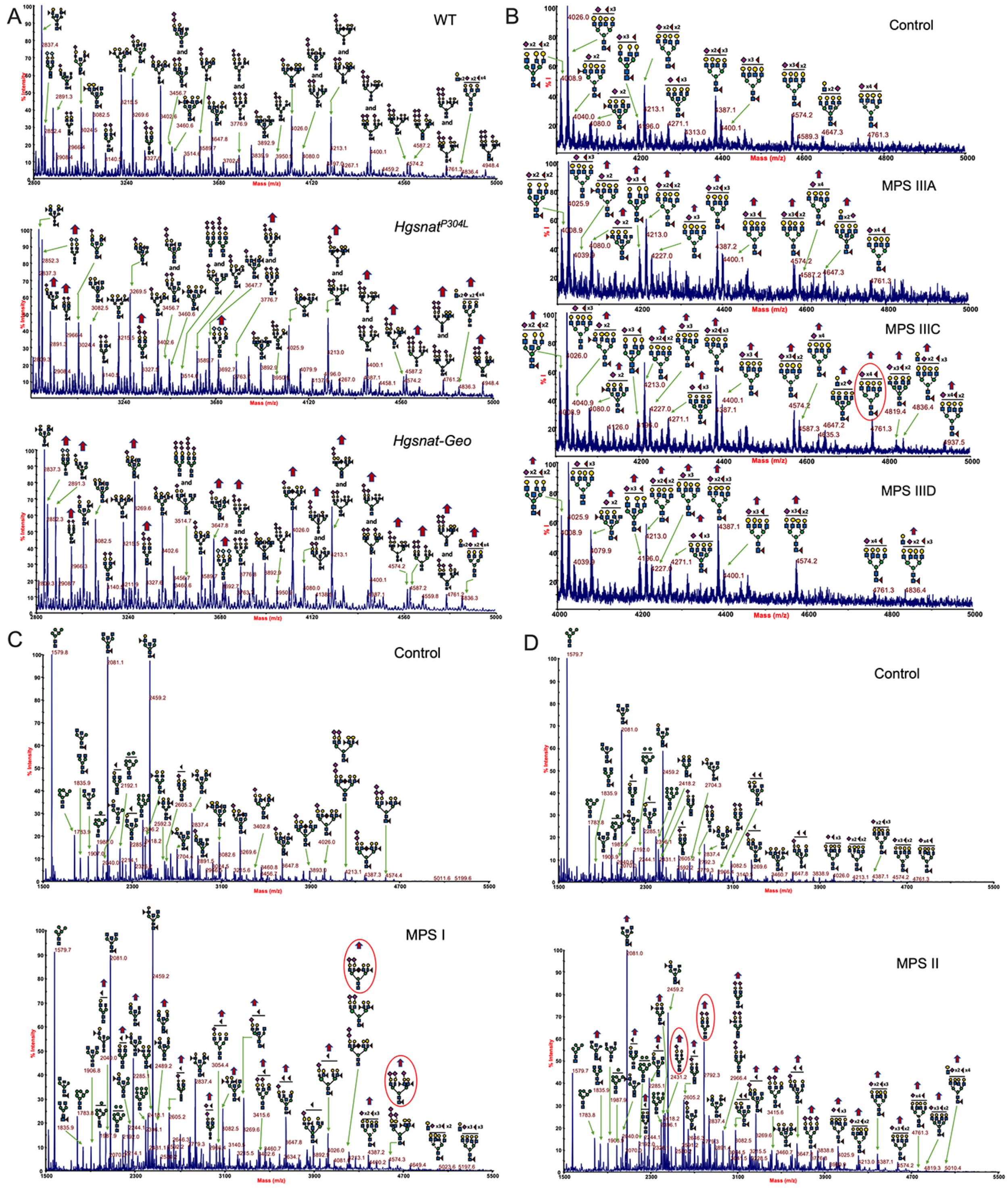

Figure S5. MALDI-TOF mass spectrometry analysis shows increased sialylation of brain proteins in MPS mouse models and human MPS patients

(A) Sialylation of N-linked glycans of brain glycoproteins from 10-month-old control, *Hgsnat*^{P304L} and *Hgsnat-Geo* mice was analyzed by MALDI-TOF MS. Red arrows mark glycan species

containing one or more sialic acid residues with increased intensity for MPS IIIC animals. Each spectrum shows a mass range between m/z 2800 and 5500.
(B) Sialylation of N-linked glycans from brain glycoproteins of MPS IIIA, IIIC and IIID patients, as well as age and sex-matched controls, was analyzed as in **A**. Mass spectra in the mass range between *m/z* 4000 and 5000 m/z show an increased intensity for sialylated glycan species for MPS III patients (red arrows).
(C) Sialylation of N-linked glycans from brain glycoproteins of MPS I patient, as well as age and sex-matched control, was analyzed as in **A**. Mass spectra in the range between *m/z* 1500 to 5000 show increased levels of sialylated glycan species for MPS I patients compared to controls (red arrows). Spectra also show a widespread increase in fucosylation together with major increase of glycans bearing sialyl-lewis epitopes (red circles), typically not present in human brain N-glycans.
(D) Sialylation of N-linked glycans from brain glycoproteins of neurological MPS II patient, as well as age and sex-matched control, was analyzed as in **A**. Mass spectra in the range between *m/z* 1500 to 5000 show increase in sialylated and fucosylated species (red arrows) in MPS II patients. This includes two biantennary unfucosylated N-sialoglycans (circled in red), usually present at a very low level in the human brain N-glycome.
GlcNAc, blue square; Man, green circle; Gal, yellow circle; Neu5Ac, purple diamond; Neu5Gc, light blue diamond; Fuc, red triangle.

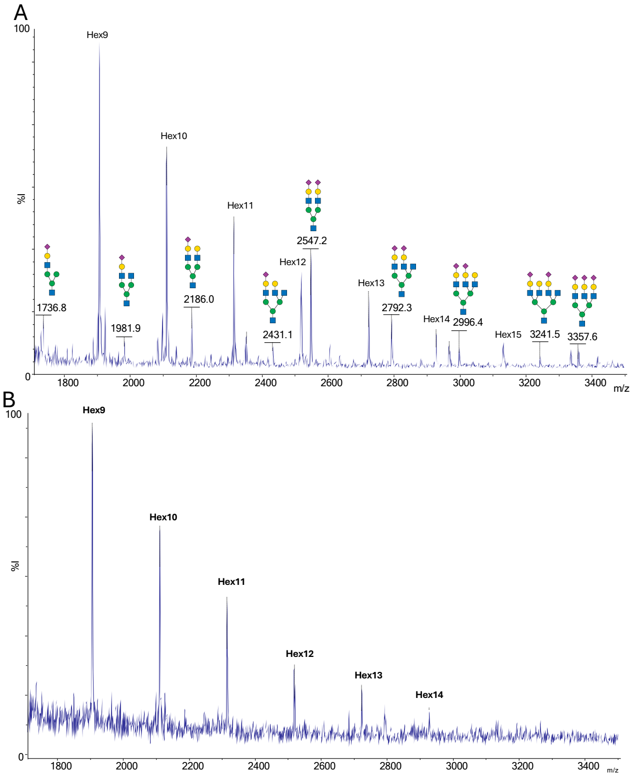

Figure S6. MALDI-TOF mass spectrometry reveals the presence of urinary sialylated glycans in MPS IIIC patients.

Representative MALDI-TOF MS profile of permethylated urinary free glycans in the mass range between *m/z* 1700 and 3500 from a MPS IIIC patient **(A)** and sex/age matching healthy control **(B)**. Beside a polyhexose ladder frequently occurring as contaminant of urine samples, samples from MPS IIIC patients contain sialylated glycans not present in the urine of a healthy control. N-acetylglucosamine, blue square; mannose, green circle; galactose, yellow circle; sialic acid, purple diamond.

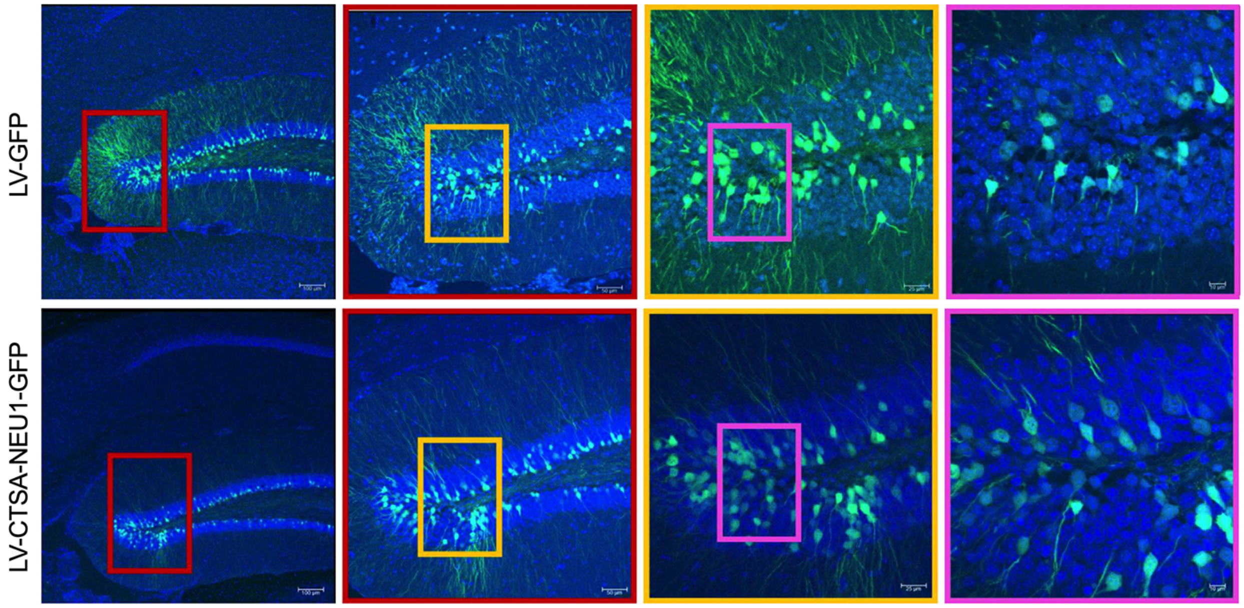

Figure S7. Expression of GFP and NEU1-GFP proteins in hippocampus and dentate gyrus of mice treated with stereotaxic injections of LV-GFP and LV-CTSA-IRES-NEU1-GFP.

GFP and NEU1-GFP proteins show high levels of expression in the neurons of hippocampus and dentate gyrus of mice six months after stereotaxic injections of LV-GFP and LV-CTSA-IRES-NEU1-GFP, respectively. Panels show representative confocal fluorescent images of hippocampus captured at different magnification. GFP-mediated fluorescence is shown in green. Nuclei were counterstained with DAPI (blue). Boxes show positions of zoomed areas. Scale bars: 100 μm and 50, 25 and 10 μm in zoomed images.

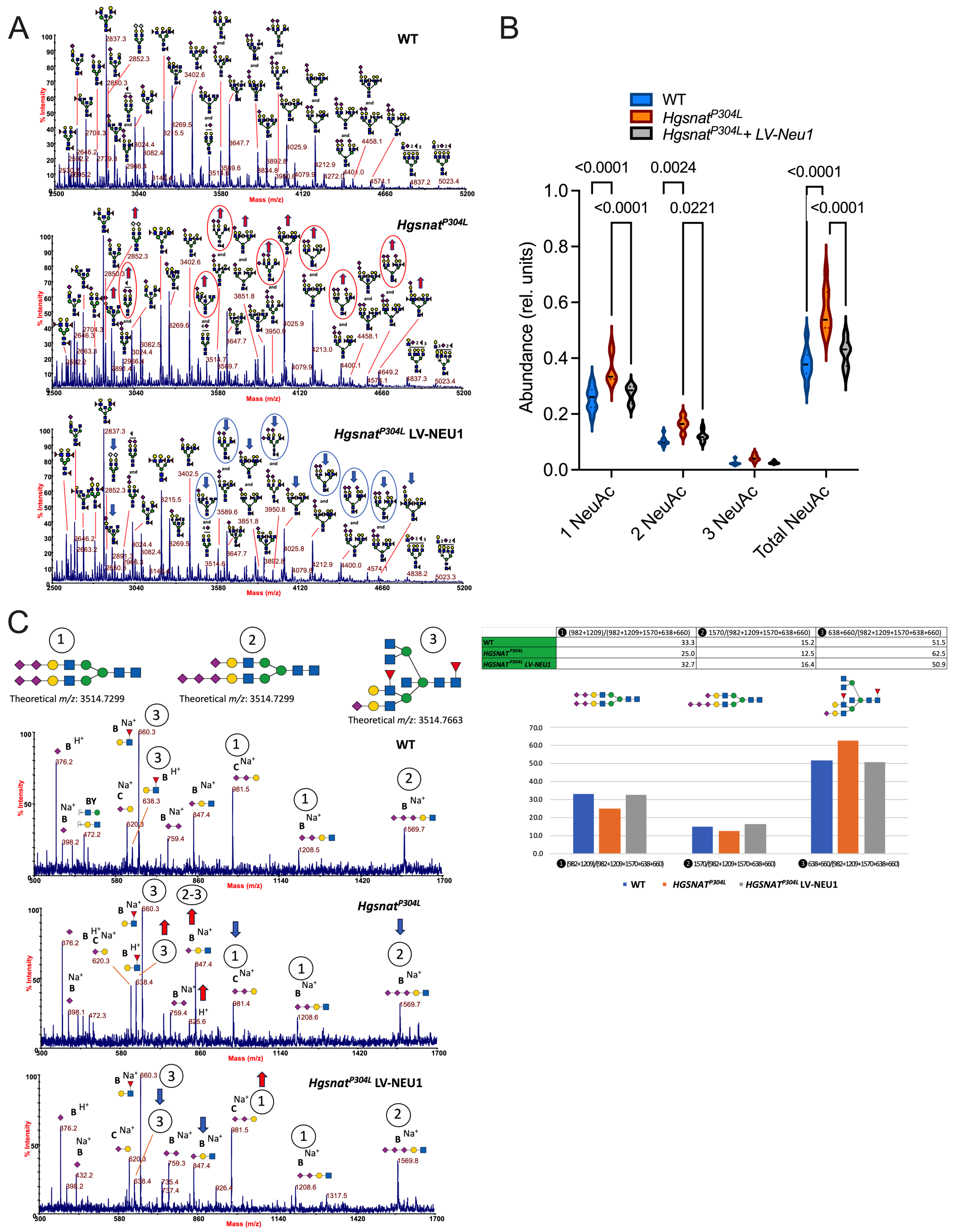

	❶ (982+1209)/(982+1209+1570+638+660)	❷ 1570/(982+1209+1570+638+660)	❸ 638+660/(982+1209+1570+638+660)
WT	33.3	15.2	51.5
$HGSNAT^{P304L}$	25.0	12.5	62.5
$HGSNAT^{P304L}$ LV-NEU1	32.7	16.4	50.9

Figure S8. MALDI-TOF mass spectra show restored sialylation of hippocampal proteins in *Hgsnat*^{P304L} mice treated with LV-CTSA-IRES-NEU1-GFP

(A) Sialylation of N-linked glycans of hippocampal glycoproteins from 6-month-old WT, *Hgsnat*^{P304L} and *Hgsnat*^{P304L} mice treated with LV-CTSA-IRES-NEU1-GFP (LV-NEU1) was

analyzed by MALDI-TOF MS. Mass-spectra in the range between *m/z* 2500 to 5200 show increased levels of sialylated glycan species for untreated *Hgsnat*^{P304L} compared to WT mice (red arrows) and reduced levels of sialylated glycans for LV-CTSA-IRES-NEU1-GFP treated mice compared to untreated *Hgsnat*^{P304L} mice (blue arrows). Red circles mark the sialylated glyco-isomers that are enhanced in *Hgsnat*^{P304L} mice and blue circles the ones reduced after the treatment according to the MS/MS analysis. **(B)** Box and violin plot show average relative intensity of MS peaks of glycan species containing 1, 2 or 3 NeuAc residues or relative intensity of all sialylated glycans. P values were calculated by 2 way ANOVA with Holm-Šídák's multiple comparisons test. N=9 per group. **(C)** Representative MS/MS analysis of the peak at *m/z* 3514.7 (low mass-range, between *m/z* 300 and 1700) shows that this species represent a mixture of three unique glycans with different positions of NeuAc moieties. Comparison of the relative intensity of the ions from non-reducing terminals revealed that fragments containing NeuAc 2,3 or 2,6-linked to Gal are increased in untreated and reduced in LV-CTSA-IRES-NEU1-GFP treated *Hgsnat*^{P304L} compared to WT mice, while the intensity of fragment ions with 2,8-linked NeuAc dimers or trimers follows the opposite trend; they reduced in untreated *Hgsnat*^{P304L} and return to WT levels in treated mice. This is reflected in the relative abundance of the three isomers at *m/z* 3514.7, as shown in the bar graph.
GlcNAc, blue square; Man, green circle; Gal, yellow circle; Neu5Ac, purple diamond; Neu5Gc, light blue diamond; Fuc, red triangle.

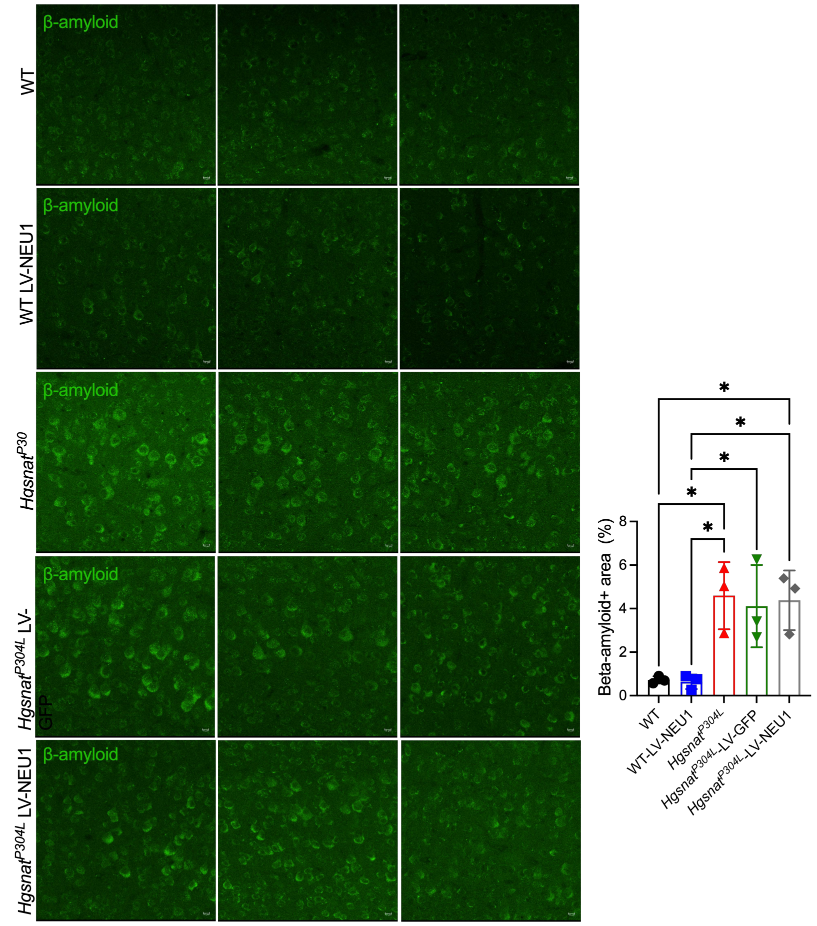

Figure S9. ***Hgsnat*^{P304L} mice, treated with LV-CTSA-IRES-NEU1-GFP, show amyloid protein accumulation in level 4 and 5 pyramidal cortical neurons similar to that in *Hgsnat*^{P304L} and sham-treated *Hgsnat*^{P304L} mice.**

Representative confocal images of the somatosensory cortex of **(A)** WT mice, **(B)** WT mice injected with LV-CTSA-IRES-NEU1-GFP (LV-NEU1), **(C)** *Hgsnat*^{P304L} mice, **(D)** *Hgsnat*^{P304L} mice injected with LV-GFP, and **(E)** *Hgsnat*^{P304L} mice injected with LV-CTSA-IRES-NEU1-GFP. Brain sections were stained with antibodies against beta-amyloid protein (green). Confocal images were taken using a 40x objective, scale bar: 25 µm. Data show means ± SD, n=5. *P < 0.05, determined by one-way ANOVA with Tukey post hoc test.

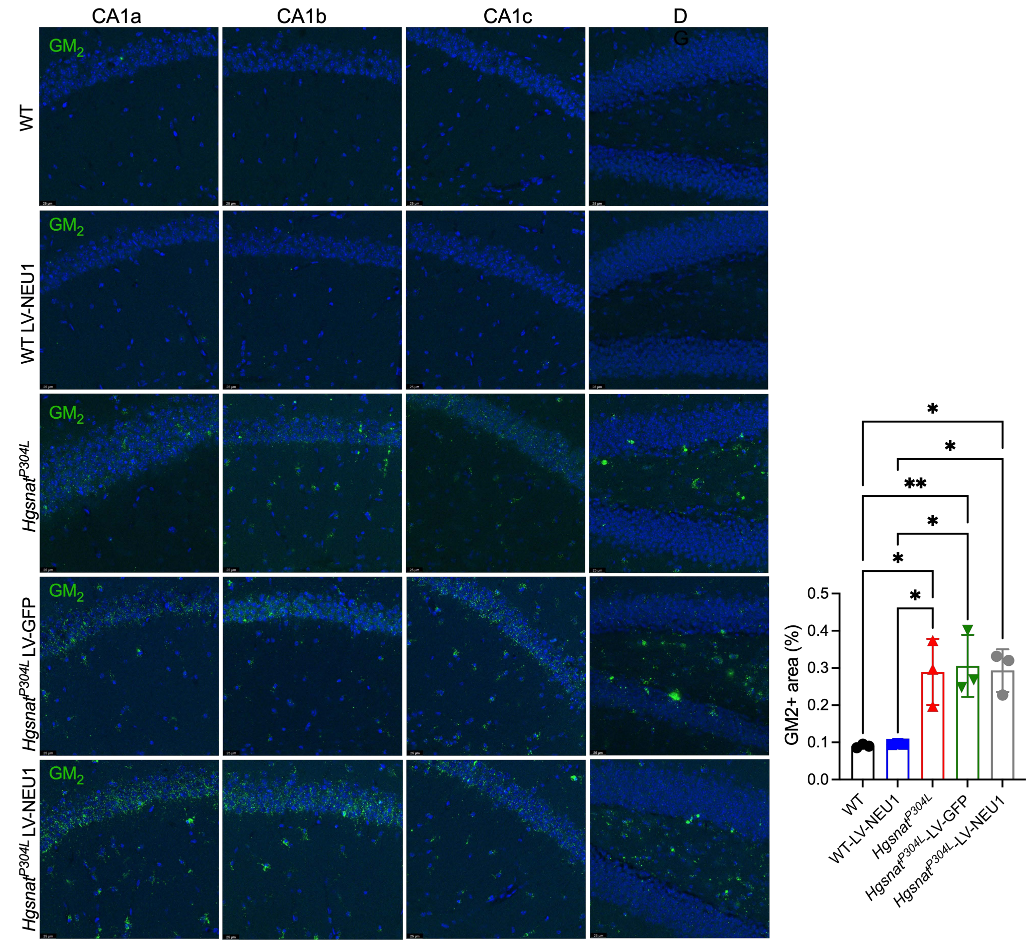

Figure S10. *Hgsnat*^{P304L} mice treated with LV-CTSA-IRES-NEU1-GFP show GM2 ganglioside accumulation in pyramidal hippocampal neurons similar to that in *Hgsnat*^{P304L} and sham-treated *Hgsnat*^{P304L} mice.

Representative confocal images of four different regions of the hippocampus of **(A)** WT mice, **(B)** WT mice injected with LV-CTSA-IRES-NEU1-GFP (LV-NEU1), **(C)** *Hgsnat*^{P304L} mice, **(D)** *Hgsnat*^{P304L} mice injected with LV-GFP, and **(E)** *Hgsnat*^{P304L} mice injected with LV-CTSA-IRES-NEU1-GFP. Brain sections were stained with antibody against GM2 ganglioside (green). The nuclei were counterstained with DAPI (blue). Confocal images were taken using a 40x objective, scale bar: 25 µm. Data show means (± SD), n=3. *P < 0.05, **P < 0.01, determined by one-way ANOVA with Tukey post hoc test.

Table S1. Human MPS patients and non-MPS controls used in the study

Identification	Disorder	Cause of Death	Age	Sex	Ethnicity
662	Control	Accident, multiple injuries	12	Female	White
754	Control	Asthma	11	Female	Native Hawaiian or Other Pacific Islander
1266	Control	ASCVD (Arteriosclerotic Cardiovascular Disease)	42	Male	White
4641	Control	Asthma	24	Female	Black or African-American
5287	Control	Accident, multiple injuries	23	Female	White
5813	Control	ASCVD (Arteriosclerotic Cardiovascular Disease)	20	Male	Black or African-American
5977	Control	Smoke inhalation	6	Female	White
561	MPS I	Complications of disorder	6	Female	White
902	MPS II	Complications of disorder	42	Male	White
HBCB_18_01_OC	MPS II	Unknown	13	Male	White
-3617	MPS IIIA	Complications of disorder	12	Female	White
563	MPS IIIA	Complications of disorder	11	Female	White
6194	MPS IIIC	Complications of disorder	20	Male	Black or African-American
5411	MPS IIID	Complications of disorder	24	Female	White
5424	MPS IIID	Complications of disorder	23	Female	White

Table S2. Structures of N-linked glycans from iPSC-derived cortical neurons

P = Paucimannose; O = Oligomannose; H = Hybrid; C = Complex; B = Bisected; F = Fucosylated; S = Sialylated

	Theoretical *m/z*	Structure	Glycan type
1	1345.67	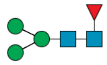	P- F
2	1375.68	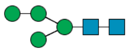	P
3	1579.78	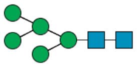	O
4	1783.88	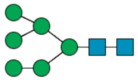	O
5	1835.92	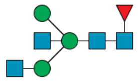	C - B - F
6	1906.96	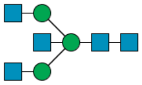	C - B
7	1987.98	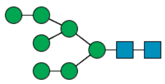	O
8	2040.02	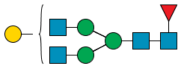	C - F
9	2081.05	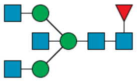	C -B - F
10	2192.08	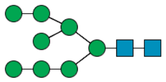	O
11	2244.12	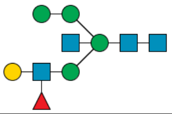	H - B - F
12	2285.15	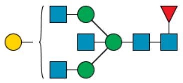	C - B - F
13	2326.18	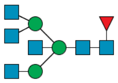	C - B - F
14	2360.18	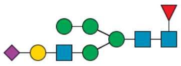	H - F
15	2396.27	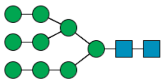	O
16	2459.24	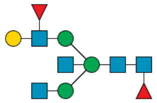	C - B - F
17	2489.25	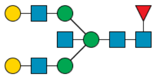	C -B - F
18	2530.28	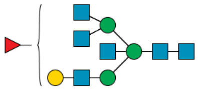	C -B - F
19	2564.27	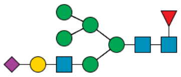	H - B- F- S
20	2600.28	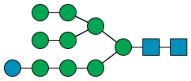	O
21	2605.30	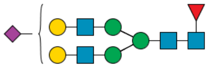	C - F - S
22	2646.32	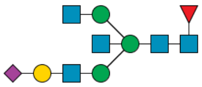	C - B - F -S
23	2704.37	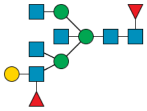	C - B - F -S
24	2779.39	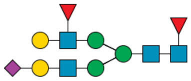	C - F - S
25	2837.43	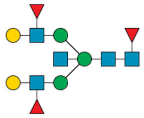	C - B - F
26	2850.43	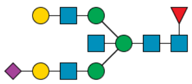	C - B - F -S
27	2891.45	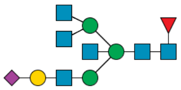	C - B - F -S
28	2966.47	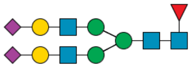	C - F - S
29	3024.51	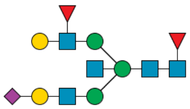	C - B - F -S

	Theoretical *m/z*	Structure	Glycan type
**30**	3054.52	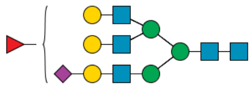	**C - F -S**
**31**	3082.55	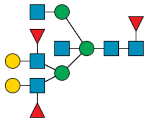	**C - B -F**
**32**	3095.55	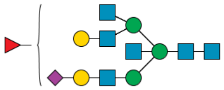	**C - B - F -S**
**33**	3211.60	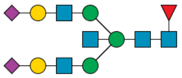	**C - B - F -S**
**34**	3269.64	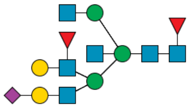	**C - B - F -S**
**35**	3415.70	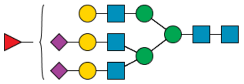	**C - F -S**
**36**	3456.72	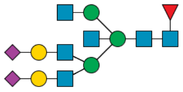	**C - B - F -S**
**37**	3514.77	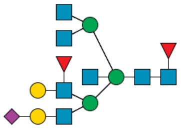	**C - F - S**
**38**	3589.79	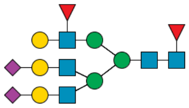	**C - F - S**
**39**	3646.83	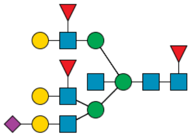	**C - F - S**
**40**	3776.87	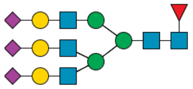	**C - F - S**
**41**	3834.91	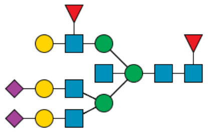	**C - B - F -S**

	Theoretical *m/z*	Structure	Glycan type
**42**	3864.92	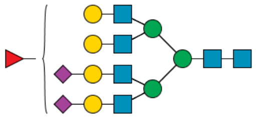	**C - F -S**
**43**	3892.95	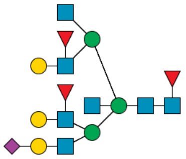	**C - B - F -S**
**44**	4022.00	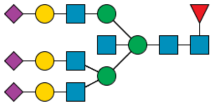	**C - B - F -S**
**45**	4026.02	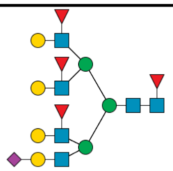	**C - F -S**
**46**	4039.01	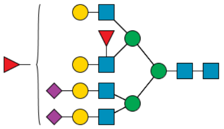	**C - F -S**
**47**	4213.10	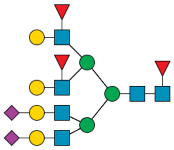	**C - F -S**
**48**	4226.10	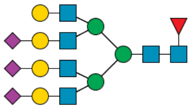	**C - F - S**
**49**	4400.19	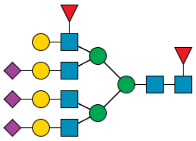	**C . F - S**

Table S3. Antibodies and their dilutions used for immunocytochemistry and immunohistochemistry.

Antigen	Host/Target Species	Dilution	Manufacturer
Heparan sulphate (HS)	Mouse anti-mouse	1:200	Amsbio (370255-S)
LAMP2	Rat anti-mouse	1:100	DSHB (ABL-93-s)
Neuraminidase (NEU1)	Rabbit anti-mouse	1:200	Abcam (ab233119)
vGLUT1	Rabbit anti-mouse	1:200 (tissues) 1:1000 (cells)	Abcam (ab104898)
PSD-95	Mouse anti-mouse	1:200 (tissues) 1:1000 (cells)	Abcam (ab99009)
Synapsin-1 (Syn1)	Rabbit anti-mouse	1:200 (tissues) 1:1000 (cells)	Abcam (ab64581)
β-amyloid (D54D2)	Rabbit anti-mouse	1:200	Cell Signalling (8243S)
G_{M2}	Mouse humanized	1:300	KM966
MAP2	Chicken anti-mouse	1:500	Abcam (ab5392)
BDNF	Mouse anti mouse	1:2000	DSHB (#9-S)
Neurofilament Medium Chain	Rat anti-mouse	1:200	DSHB (2H3)
