# Supplementary material for: Neuraminidase 1 secondary deficiency contributes to CNS pathology in neurological mucopolysaccharidoses via brain protein hypersialylation
Source: J Clin Invest. 2025 Jun 17;135(16):e177430. doi: 10.1172/JCI177430 (PMC12352893; doi:10.1172/JCI177430)
Supplement: Unedited blot and gel images [file jci-135-177430-s123.pdf]

8/8/24

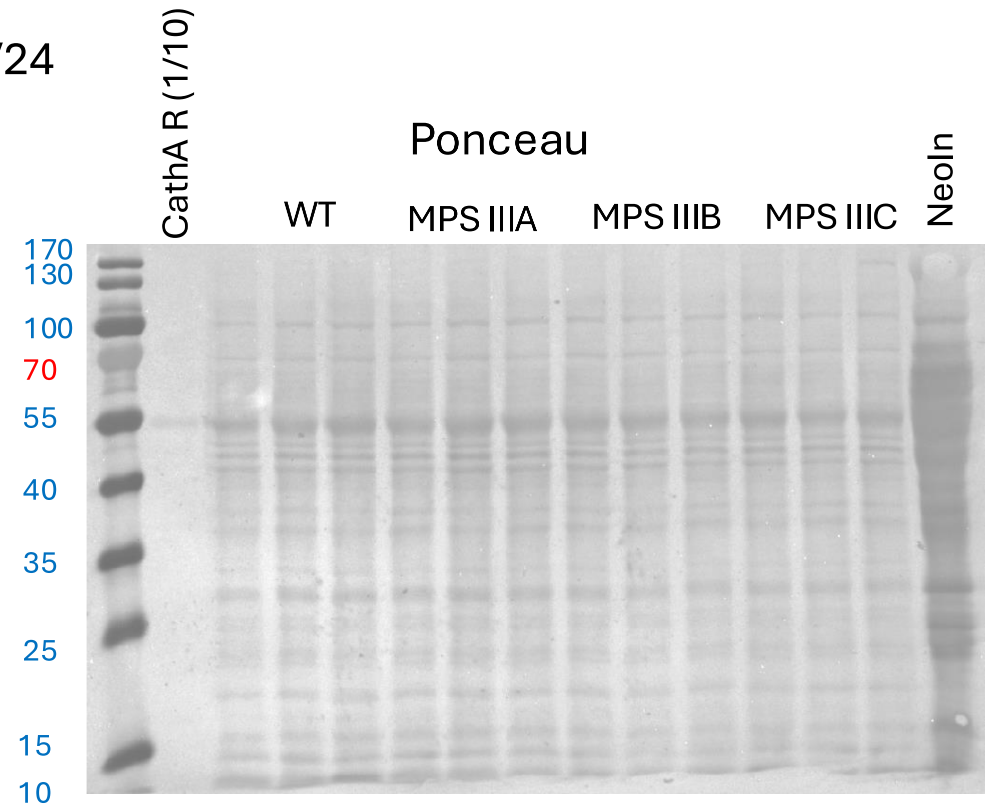

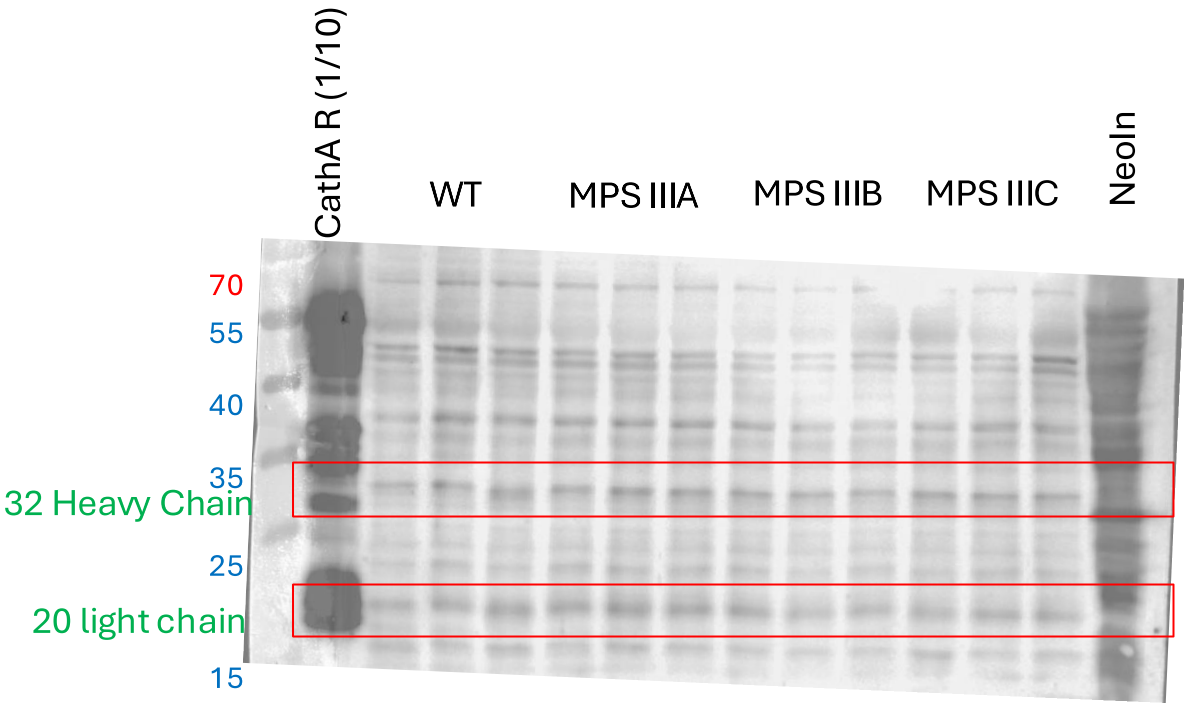

Figure: Normal expression of Cathepsin A in MPS IIIA, MPS IIIB, and MPS IIIC murine brains.
Brain samples from MPS IIIA (n=3), MPS IIIB (n=3), and MPS IIIC (n=3) mice were prepared and compared to WT mouse brains (n=3), recombinant Cathepsin A protein (dilution 1:10), and CathA NeoIn mouse brains.
The bar graph represents Cathepsin A protein expression (both heavy and light chains) normalized to total protein (mean ± SD, two-way ANOVA).

Methods

Western Blot with Concanavalin A (Con-A) Sepharose Enrichment

Brain samples (~200 mg) were homogenized with a ratio of 1:5 (w/v) in a buffer containing 50 mM sodium acetate (pH 5), 100 mM NaCl, 1 mM $CaCl_2$, 1 mM $MgCl_2$, 1 mM $MnCl_2$, and 1% zwittergent. After 2 hours of rocking at 4°C, the lysates were cleared by centrifugation at 13,300 rpm for 20 min at 4°C.

To enrich glycoproteins, concanavalin A (Con-A) Sepharose beads (Pharmacia Biotech, catalog #17-0440-01) were equilibrated in the buffer. One milliliter of supernatant was incubated with Con-A beads at 4°C overnight under gentle rotation. After centrifugation (2 min at 5,000 × g), the beads were washed three times with the buffer.

For Western blot analysis, 50 µL of 1X Laemmli buffer was added to the pellet, vortexed, boiled for 5 min, and centrifuged for 5 min at 10,000 rpm. Twenty microliters of supernatant were loaded onto a 12% SDS-PAGE gel, and proteins were separated by electrophoresis and transferred onto a nitrocellulose membrane. Membranes were blocked with blocking solution (5% BSA in TBST) for 1 hour at room temperature. Membranes were incubated overnight at 4°C with a primary anti-NEU1 rabbit monoclonal antibody (ABclonal #A25845, 1:1000 in 5% BSA/TBST), followed by incubation with an HRP-conjugated anti-rabbit IgG antibody at a 1:10,000 dilution. Signal detection was performed using ECL reagent and imaged with the ChemiDoc system (Bio-Rad). Densitometric analysis was carried out using Image Lab. Total protein normalization was performed using Ponceau Red staining.

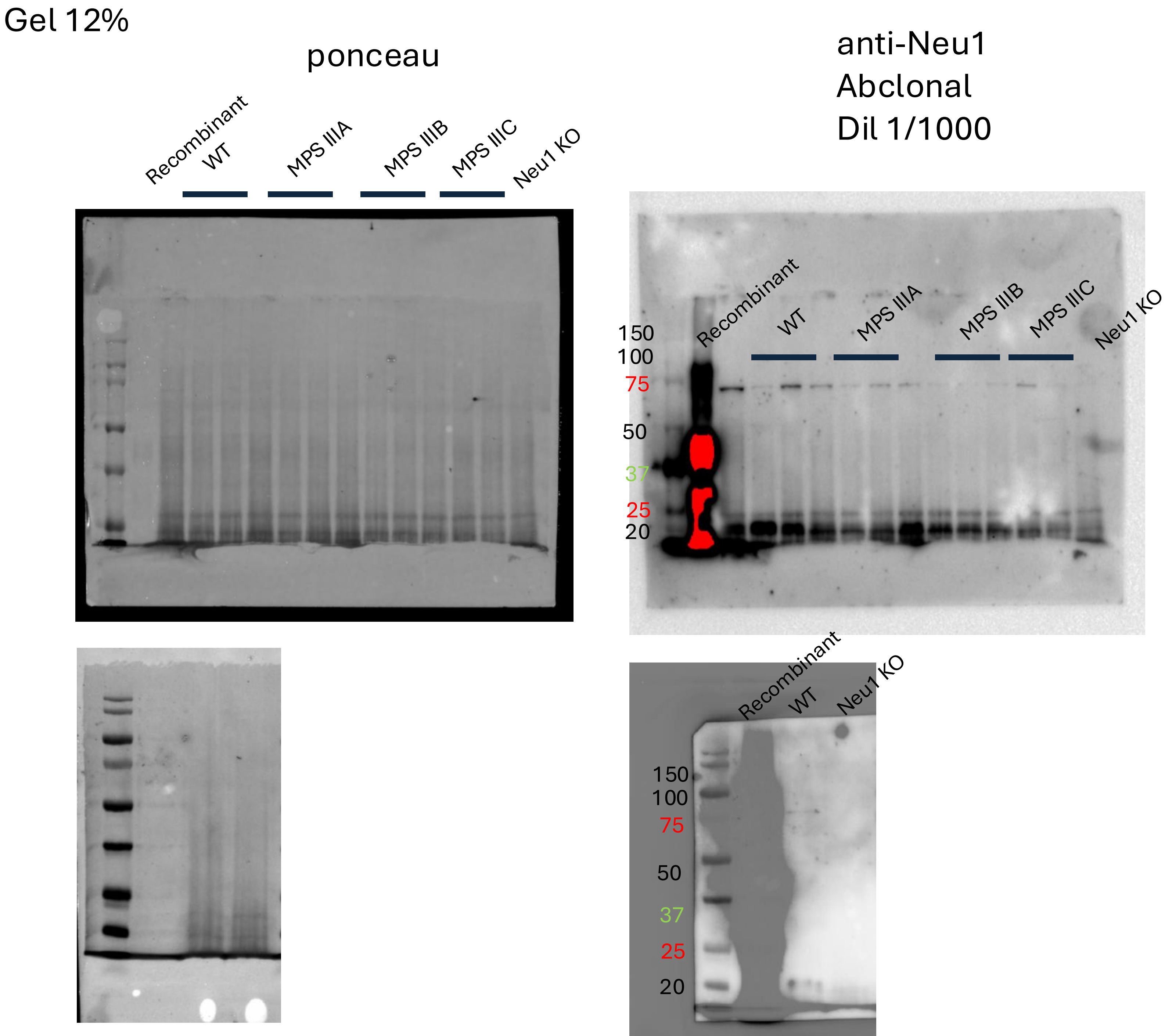

Figure: Decreased expression of neuramin
MPS IIIA, MPS IIIB, and MPS IIIC murine bra
Brain samples from MPS IIIA (n=3), MPS III
MPS IIIC (n=3) mice were prepared for wes
Concanavalin A (Con-A) Sepharose Enric
compared to WT mouse brains (n=3), reco
protein (dilution 1:50), and Neu1 KO mous
The bar graph represents Neu1 protein exp
(70kDa) normalized to total protein (mean
ANOVA).

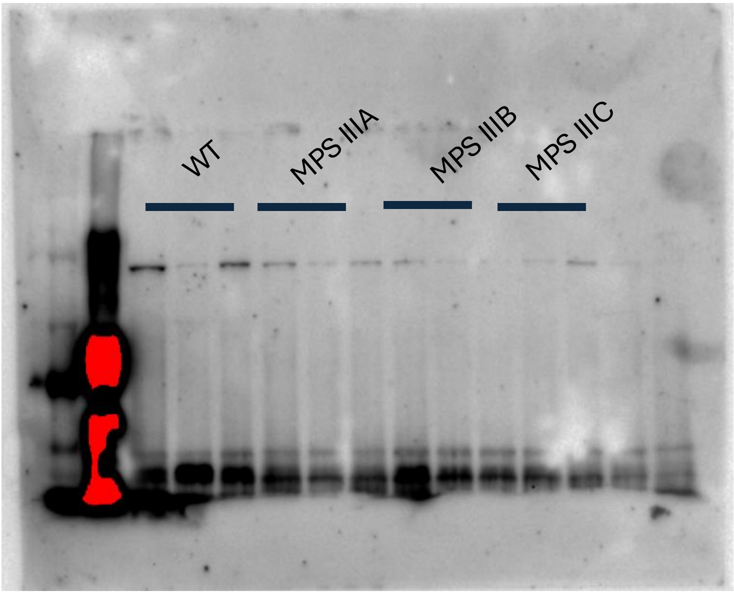

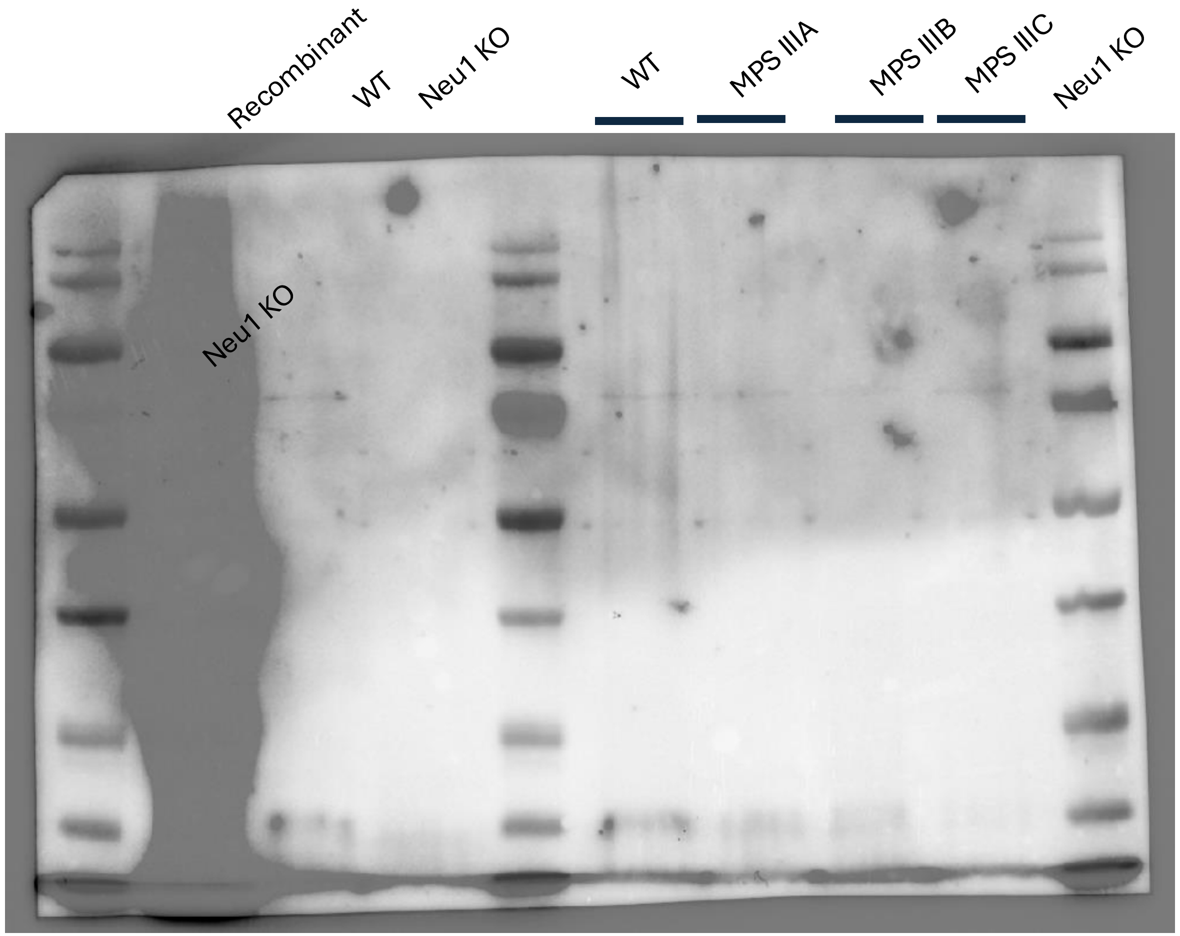

Other images of the same membranes
